# Novel *Plasmodium* antigens identified via genome-based antibody screen induce protection associated with polyfunctional T cell responses

**DOI:** 10.1038/s41598-017-15354-0

**Published:** 2017-11-08

**Authors:** Sophie Schussek, Angela Trieu, Simon H. Apte, John Sidney, Alessandro Sette, Denise L. Doolan

**Affiliations:** 1 QIMR Berghofer Medical Research Institute, Infectious Diseases Programme, Herston, QLD 4006 Australia; 2 University of Queensland, School of Medicine, Herston, QLD 4006 Australia; 3 La Jolla Institute of Allergy and Immunology, San Diego, CA 92121 USA; 4 Centre for Biosecurity and Tropical Infectious Diseases, Australian Institute of Tropical Health and Medicine, James Cook University, Cairns, QLD 4879 Australia

## Abstract

The development of vaccines against complex intracellular pathogens, such as *Plasmodium spp*., where protection is likely mediated by cellular immune responses, has proven elusive. The availability of whole genome, proteome and transcriptome data has the potential to advance rational vaccine development but yet there are no licensed vaccines against malaria based on antigens identified from genomic data. Here, we show that the *Plasmodium yoelii* orthologs of four *Plasmodium falciparum* proteins identified by an antibody-based genome-wide screening strategy induce a high degree of sterile infection-blocking protection against sporozoite challenge in a stringent rodent malaria model. Protection increased in multi-antigen formulations. Importantly, protection was highly correlated with the induction of multifunctional triple-positive T cells expressing high amounts of IFN-γ, IL-2 and TNF. These data demonstrate that antigens identified by serological screening are targets of multifunctional cellular immune responses that correlate with protection. Our results provide experimental validation for the concept of rational vaccine design from genomic sequence data.

## Introduction

With almost half of the world’s population (an estimated 3.2 billion people) at risk of malaria, approximately 200 million reported clinical cases and half a million deaths annually, mostly in children under the age of 5 years, malaria remains a major public health problem^1^. Due to the rapidly developing resistance of the *Plasmodium* parasites and *Anopheles* vectors to chemical interventions, vaccination against malaria is considered a public health priority. However, the complexity of the pathogen and of the immune response required to protect the human host from infection or severe disease has hindered vaccine development.

*Plasmodium spp*. parasites have a multi-stage lifecycle including an invertebrate and a vertebrate host and express ~5,300 proteins in a stage-specific manner^2^. The pre-erythrocytic stage of the parasite lifecycle represents an attractive target for vaccination, because inhibition of parasite growth in hepatocytes can reduce or complete ablate blood stage parasitemia, thus delaying or preventing clinical symptoms and disease pathology and subsequent transmission. Indeed, infection with radiation attenuated *Plasmodium* spp. sporozoites (RAS), whose development is halted in the liver, can induce sterile protection against sporozoite challenge in rodent and human models^3,4^. Attenuation of parasites has been also achieved using reverse-genetic methods to generate genetically attenuated parasites (GAP) able to invade but only partially develop within the host^5^. However, these approaches require the generation of large numbers of sporozoites in mosquitoes and there is evidence of attenuated parasites reverting to their actively infective form^6^. The success of vaccination with *P. falciparum* RAS^7^ demonstrates the feasibility of inducing sterile infection-blocking protective immunity and suggests that antigens expressed by the liver stage parasite would be effective vaccine targets. However only a very small fraction (<0.5%) of the *Plasmodium spp*. proteome has been evaluated for vaccine efficacy^8^ and antigens associated with solid sterile infection-blocking immunity have not been identified^9^. Indeed, failure to develop an effective malaria vaccine is likely due to the limited list of potential antigens thus far evaluated and their assessment predominantly as single-antigen based vaccines^10,11^.

The most advanced malaria vaccine candidate, RTS,S (also known as Mosquirix^TM^), is based on the dominant sporozoite surface protein, *P. falciparum* circumsporozoite protein (CSP)^12^, and has been in development since the 1980s. Despite many decades of research and almost 50 clinical studies, the recent Phase 3 trial of RTS,S/AS01 in Africa has been disappointing^13^. Vaccine efficacy against clinical malaria in the target group of infants aged 6–12 weeks at first vaccination was only 18% following the 3-dose primary schedule and 26% following a booster dose at 18 months, and decayed rapidly^13,14^. Of concern, this Phase 3 evaluation identified a risk of febrile seizures for RTS,S/AS01 as well as significantly more cases of both cerebral malaria and meningitis in vaccine recipients than controls and significantly higher all-cause mortality in girls^13^. Thus, although the RTS,S/AS01 vaccine received a positive scientific opinion from the European Medicines Agency for active immunization against malaria of children aged 6 weeks to 17 months^15^, the World Health Organization has not recommended RTS,S/AS01 for routine use, pending resolution of a number of uncertainties related to vaccine impact and vaccine safety as well as programmatic aspects^16^.

In order to meet the public health need and the goals of the roadmap set by the malaria vaccine community^17^, one approach being advanced by a number of researchers and funding agencies is to enhance the efficacy of RTS,S by identifying novel antigens that work in synergy with CSP. Other well characterised *P. falciparum* sporozoite and liver antigens include sporozoite surface protein (SSP2/TRAP), liver stage antigen 1 (LSA1), cell-traversal protein for ookinetes and sporozoites (CelTOS), sporozoite threonine asparagine rich protein (STARP) and exported protein 1 (EXP1) but none of these have proved to be highly efficacious in clinical studies (reviewed in ref.^8^).

An alternative strategy is to specifically avoid building on RTS,S^11^. This is based on the premise that rational antigen selection is important and that antigens such as the CSP may be “red herrings” presented by the *Plasmodium* parasite to the human host to divert responses away from other potential targets of protective immune responses. This approach is enabled by recent advances in gene sequencing and high-throughput analysis which provide the foundation for mining whole genome and proteome datasets to identify potential vaccine candidates^9,18^. We have hypothesised that proteins identified from genome-based datasets using biologically relevant criteria, such as association with protection, may be excellent vaccine targets^9^. In particular, we proposed that protein microarrays expressing the proteome of the pathogen of interest and probed with sera/plasma from exposed or immune individuals represent a promising platform to discover target antigens for vaccines and diagnostics^9,18^. In a previous study^9^, we probed a partial *P. falciparum* protein microarray with plasma from clinically divergent groups of individuals immunised with RAS, and identified 16 novel antigens strongly associated with sterile protective immunity (RAS ‘signature’ antigens). In another study^19^, we utilised the same protein microarray platform to screen plasma from children and young adults living in endemic areas of Mali and identified 46 proteins associated with protection from disease during the malaria season (naturally acquired immunity ‘signature’ antigens).

In the work presented herein, we have build on our previous studies to investigate four antigens associated with protection in humans for their vaccine potential, in the virulent *P. yoelii* rodent malaria model. We evaluated T cell and antibody responses as well as protection against sporozoite and blood-stage challenge induced by immunisation of BALB/c mice with the *P. yoelii* orthologues of these novel antigens, both individually and in combination, using DNA/protein and peptide/adjuvant regimens. The first strategy represents a whole-antigen immunisation strategy incorporating immunisation with antigen encoding plasmid DNA and recombinant protein in order to maximise immune responses to all possible epitopes; DNA/protein strategies have previously proved successful in inducing protection in animal models of malaria^20^. The peptide/adjuvant strategy, on the other hand, was designed to focus immune responses on putative CD4^+^ and CD8^+^ T cell epitopes predicted to bind BALB/c MHC class I and class II molecules with high affinity. We elected to formulate the pool of predicted peptide epitopes with the adjuvant AbISCO100, a commercially available ISCOM matrix formulation, previously described to generate a balanced immune response with both Th1 and Th2 characteristics^21^. We show that each of the four proteins is a target of protective immune responses and correlate protection with the induction of multifunctional Th1 T cell responses. We further show enhanced efficacy with multi-antigen combinations, and correlate vaccine-induced protection with the induction of multifunctional triple-positive T cells expressing high amounts of IFN-γ, IL-2 and TNF.

## Results

### Antigen selection & bioinformatic analysis

The selection of novel genome-derived antigens for evaluation *in vivo* was based on the results of two protein microarray studies. In one study, 16 pre-erythrocytic proteins were associated with protection induced in human volunteers by immunisation with radiation attenuated *P. falciparum* sporozoites (RAS ‘signature’ antigens)^22^. Four of these 16 novel proteins (MAL13P1.22, PFI0925w, PFL1620w and PFE0060w) were also identified in another study as associated with protection against re-infection in individuals living in endemic areas in Mali (naturally acquired immunity ‘signature’ antigens)^19^ (Table 1). Thus, the four proteins each shared signatures implicated with protection induced by both experimental immunisation and natural infection. One of these, *P. falciparum* erythrocyte surface protein 2 (PIESP2; PFE0060w), does not have any known orthologues in rodent malaria species (Table 1). Antigen (PF14_0051) was also highly associated with protection in RAS immunised volunteers and reactive in protein microarray studies of multiple naturally exposed populations from different endemic regions.

**Table 1:** Characteristics of target antigens.

*P.y* gene ID	*P.f* ortholog		Product description	Signature ranking		*P.f* MS data^c^				SP^d^	TM^d^	SNP densitye	BLASTP identity to human^f^	Most immunogenic fragment^g^	*P.y* transcript size	*P.y* immunogenic fragment size
				IrrSpz^a^	Naturally acquired^b^	Spz	Mrz	Tpz	Gmt							
PY01533	PF3D7_1304100	MAL13P1.22	DNA ligase I	20	24	2	0	2.2	0	yes	0	0.01	47%, 69%	full-length	2481	2481
PY01606	PF3D7_0918900	PFI0925w	gamma-glutamylcysteine synthetase	1	18	0	4.4	0	0	no	0	0.005	36%, 55%	full-length	3000	3000
PY03673	PF3D7_1405400	PF14_0051	DNA mismatch repair protein, putative	3	N/A	1.3	1.3	0	0	yes	0	0.019	26%, 9%	exons 2+4	4282	1647
PY03832	PF3D7_1233600	PFL1620w	asparagine/aspartate rich protein, putative	5	10	0.2	0	0	0	no	0	0.016	34%, 4%	exon 1 segment 3	9161	2000
PY03168	PF3D7_0304600	PFC0210c	circumsporozoite protein	14	N/A	10.1	0	0	0	yes	1	0.055	33%, 45%	full-length	1176	1176

^a^‘Signature antigens’ associated with protection after experimental vaccination with radiation-attenuated sporozoites (RAS)^22^. ^b^‘Signature antigens’ associated with protection against severe malaria in children and young adults in Mali^19^. ^c^Multidimensional protein identification technology to survey of sporozoite (Spz), merozoite (Mrz), trophozoite (Trp) and gametocyte (Gmt) proteome^30^. ^d^SP, signal peptide; and TM, transmembrane regions of *P. falciparum* proteins, and *P. yoelii* orthologues, using SignalP^28,29^ and TMHMM2^27^. ^e^Single nucleotide polymorphism (SPN) density calculated as number of total SNP over the gene length in bp (both derived from PlasmoDB; www.plasmodb.org). ^f^BLASTP identity of target proteins to human proteome and query coverage^87^. ^g^Most immunogenic *P. falciparum* open reading frame fragment identified via protein microarray studies^22^.

The protein microarray used to identify these antigens was fabricated using a high-throughput cloning and protein expression system^22–24^. In that system, the size of target gene fragments is limited to approximately 3000 bp due to reduced PCR amplification efficiency^23^. Accordingly, large proteins were split into multiple segments for evaluation in the *P. yoelii* rodent model. Two of our four target antigens could be evaluated using the full-length orthologous genes (PY01533: 2481 bp, PY01606: 3000 bp), while the other two (PY03673: 4282 bp, PY03832: 9161 bp) were split into antigenic segments corresponding to the *P. falciparum* sequence showing the highest reactivity on the protein microarrays (exon 1 segment 3 for PY03832; exons 2 and 4 for PY03673) (Table 1).

Mass spectrometry data for *P. falciparum* sporozoite and blood-stages showed expression of the four target antigens in sporozoites, as well as blood-stage merozoites or blood-stage trophozoites^25^ PlasmoDB; www.plasmoDB.org). Proteomic and transcriptomic datasets of *P. yoelii* sporozoite and liver stages indicated that PY01533, PY1606 and PY03673 are most highly expressed in salivary gland sporozoites and PY03832 in liver-stage parasite 40 hr after infection (Table 2) (www.plasmoDB.org, ref.^26^).

**Table 2:** Bioinformatic analysis of target antigens.

*P.y* gene ID	immunogenic fragment^g^	% homology of *P.y* to other *P*. species^a^					highest protein expression in *P.y* ^a^	BLASTP *P.y* to mouse %identity, %coverage	Vaxijen^b^	Algpred^c^				TargetP1.1^d^
		*P.f*	*P.b*	*P.ch*	*P.k*	*P.v*			antigen probability	IgE epitope	MAST	SVM score	ARPs	Subcellular location
PY01533	full-length	78.5	73.5	75	74.6	75.4	sporozoite, blood-stage schizont	50%, 82%	0.5072	VKGEEKEPSK	Non allergen	1.13	no hits	n.d.
PY01606	full-length	64.3	66.1	63.9	67	62.7	sporozoite, blood-stage schizont	29%, 68%	0.5979	no	Non allergen	1.12	no hits	n.d.
PY03673	full-length	48.7	51.1	53.5	56.2	47.4	salivary sporozoite, liver-stages	28%, 9%	0.4744	no	Non allergen	1.03	no hits	mitochondrial
PY03673	exons 2+4	42.2	58.6	57.6	38.6	41.2								
PY03832	full-length	32.6	44.3	28.6	26.3	24.6	liver stage 40h after infection	36%, 7%	0.6209	no	Non allergen	1.11	no hits	n.d.
PY03832	exon 1 segment 3	61.5	92.1	84.7	38.9	47.6								
PY03168	full-length	40.3	36.8		20.9	19.4	midgut sporozoite	49%, 28%	0.0558	no	Non allergen	0.31	no hits	n.d.

^a^Nucleotide ClustalW2.2 alignment of *Py* 17XNL sequences to other *Plasmodium* species: *P. falciparum* 3D7 (P.f), *P. berghei* ANKA (P.b), *P. chabaudi chabaudi* (P.ch), *P. knowlesi* strain H (P.k), *P. vivax* Sal-1 (P.v); data shown as alignment score reflecting % identity of aligned sequences; sequences and protein expression data derived from PlasmoDB (www.plasmodb.org). ^b^Alignment-free approach for antigen prediction, which is based on auto-cross covariance (ACC) transformation of protein sequences into uniform vectors of principal amino acid properties^25^. ^c^ http://www.imtech.res.in/raghava/algpred/; Prediction of allergens based on similarity of known epitopes with any region of protein, IgE epitope mapping, MEME/MAST allergen motifs, amino acid or dipeptide composition, BLAST search against 2890 allergen-representative peptides (ARPs). ^d^ http://www.cbs.dtu.dk/services/TargetP/; Based on the predicted presence of any of the N-terminal presequences: chloroplast transit peptide (**cTP**), mitochondrial targeting peptide (**mTP**) or secretory pathway signal peptide (**SP**). n.d. = not determined.

None of our target *P. falciparum* antigens have predicted transmembrane elements, as evaluated using standard transmembrane prediction algorithms^27^. SignalP predicted a signal peptide sequence for MAL13P1.22 and PF14_0051, but not for PFI0925w and PFL1620w, suggesting possible secretion of those proteins^28,29^. A comparison of single nucleotide polymorphisms (SNP) between different *P. falciparum* isolates showed low SNP density (≤0.03) for the selected antigens (Table 1) indicating all four target antigens are highly conserved between *P. falciparum* strains. Furthermore, these antigens show high conservation across *Plasmodium* species as evidenced by high percentage of homology (56% ± 15.4%) in a ClustalW alignment of orthologous nucleotide sequences (Table 2). Three of the four *P. falciparum* antigens (except PFI0925w) had low protein sequence identity to the human proteome, indicating a low propensity of induction of autoimmune reactivity (Table 1). Similarly, a sequence analysis between the *P. yoelii* orthologues and the mouse proteome revealed that PY01606 and PY03673 had very low sequence identity to the host, while PY01533 and PY03832 were more conserved across pathogen and host species (Table 2).

Other antigen characteristics were determined using bioinformatic tools available online (TargetP 1.1: http://www.cbs.dtu.dk/services/TargetP/; Algpred: http://www.imtech.res.in/raghava/algpred/; Vaxijen: http://www.ddg-pharmfac.net/vaxijen/VaxiJen/VaxiJen.html). None of the novel antigens showed allergenic potential as demonstrated by Algpred analysis (Table 2). Additionally, an algorithm determining whole protein antigenicity based on a trained alignment-independent prediction model with 70–89% accuracy was applied and results indicated that all four candidates have antigenic potential (VaxiJen threshold for random prediction >0.5^30^ (Table 2).

### Epitope prediction

In addition to using an immunisation strategy based on full-length protein or antigenic segments, we employed a T cell epitope based strategy in order to focus immune responses on potential antigen-specific T cell epitopes. Peptides were selected on the basis of high affinity binding to MHC alleles expressed by BALB/c mice (MHC class I: H-2K^d^ and H-2D^d^; MHC class II: IA^d^ and IE^d^). A consensus algorithm approach was chosen to rank predicted peptides for all five antigens according to their scores from various prediction methods (NN-align, SMM-align, combinatorial library methods) available at the Immune Epitope Database (IEDB, www.iedb.org)^31^. The predicted epitopes for each antigen (>12,000 peptides) were ranked according to their affinity to MHC class I (H-2K^d^ and H-2D^d^) and class II (IA^d^ and IE^d^). The top 1% predicted to bind with high affinity to MHC class I alleles H-2K^d^ and H-2D^d^ (1^st^ percentile of predicted 9-mer peptides) and the top 2% predicted to bind with high affinity to MHC class II alleles IA^d^ and IE^d^ (2^nd^ percentile of predicted 15-mer peptides, except for PY03832 which was the 1^st^ percentile) were selected for our studies. A pool of all putative CD8^+^ and CD4^+^ T cell epitopes predicted for each antigen was synthesised and used to immunise mice. Additionally, a pool of *Py*CSP peptides, including both previously reported and newly predicted immunodominant CD8^+^ and CD4^+^ T cell epitopes (identified using our prediction algorithm) was used as a positive control.

### Assessment of antigen-specific reduction in parasite burden

In order to assess the protective capacity of our target antigens, we determined parasite burden in the liver and in the blood after sporozoite or pRBC challenge. Protective capacity targeting pre-erythrocytic stages was assessed by qRT-PCR of liver-stage parasite burden^32^ as well as measurement of blood-stage parasitemia via FCAB assay^33^ following sporozoite challenge, in separate groups (independent experiments). To evaluate protection directed at the blood-stage of the parasite life cycle, immunised mice were challenged with pRBCs and blood-stage parasitemia was monitored via FCAB assay. Sterile protection was defined as absence of detectable blood-stage parasitemia for 30 days after infection, and partial protection was defined as greater than 25% reduction of parasite burden in the liver or in the blood as compared to non-immunised infectivity controls. A protective index was calculated as described in *Methods* (Table 3).

**Table 3:** Protective capacity against sporozoite challenge.

antigen/immunisation		Reduction of parasite burden in the liver				Reduction of parasite burden in the blood				% sterilely protected	Protection index
		group		protected		group		protected			
		#mice	mean [%]	mice [%]	mean [>25%]	#mice	mean [%]	mice [%]	mean [>25%]		
PY01533	DNA	10	0.5	30	54.5	5	0	20	72.3	0	30.8
	peptide	5	58.8	80	73.5	5	18.2	20	30.7	0	64.9
PY01606	DNA	5	48	60	77.4	4	0	0	0	0	46.4
	peptide	5	25.8	60	43.3	5	27.4	60	39.1	0	49.4
PY03673	DNA	5	0	40	43.4	5	0	0	0	0	17.4
	peptide	5	26.5	60	37.5	5	0	0	0	0	22.5
PY03832	DNA	10	55.4	60	86.4	5	20	60	60	20	87.8
	peptide	5	4.3	40	75.3	5	34.4	60	44.1	0	56.5
Com 1/4	DNA	15	81.9	100	81.9	10	75	100	75	60	156.9
	peptide	5	79.8	100	79.8	5	84.5	100	84.5	20	164.3
Com 2/3	DNA	10	51.6	70	73.6	10	51.4	80	64.3	30	103
	peptide	5	0	0	0	5	20.3	60	46.9	0	28.1
Com 1/2/3/4	DNA	5	86.7	100	86.7	5	32	60	49.6	0	116.5
	peptide	5	75.6	100	75.6	5	66.9	100	66.9	20	142.5
PyCSP	DNA	15	83.4	100	83.4	15	75.9	100	75.9	50	159.3
	peptide	5	60.5	80	72.2	5	64.2	100	64.2	40	121.9
vector		10	0	0	0	10	0	0	0	0	0
adjuvant		10	0	10	31	10	23.2	50	32.6	0	19.4

Com1/4 = PY01533 + PY03832. Com2/3 = PY01606 + PY03673. Com1/2/3/4 = PY01533 + PY03832 + PY01606 + PY03673. ^#^Mice: number of mice per group. Mean [%]: mean reduction of parasite burden across all mice tested. Mice [%]: percentage of mice with more than 25% reduction in parasite burden compared to infectivity controls. Mean [>25%]: mean reduction of parasite burden across mice with more than 25% reduction in parasite burden compared to infectivity controls. % sterilely protected: percentage of mice with complete absence of blood-stage parasitemia per group. Protection index: sum of mean reduction of parasite burden in the liver and in the blood calculated as the mean reduction of parasite burden for all protected mice per group (“mean-protected”) times the number of mice per group with more than 25% reduced parasite burden (“% mice”) compared to control group.

### Individual antigens

**Figure 1:**
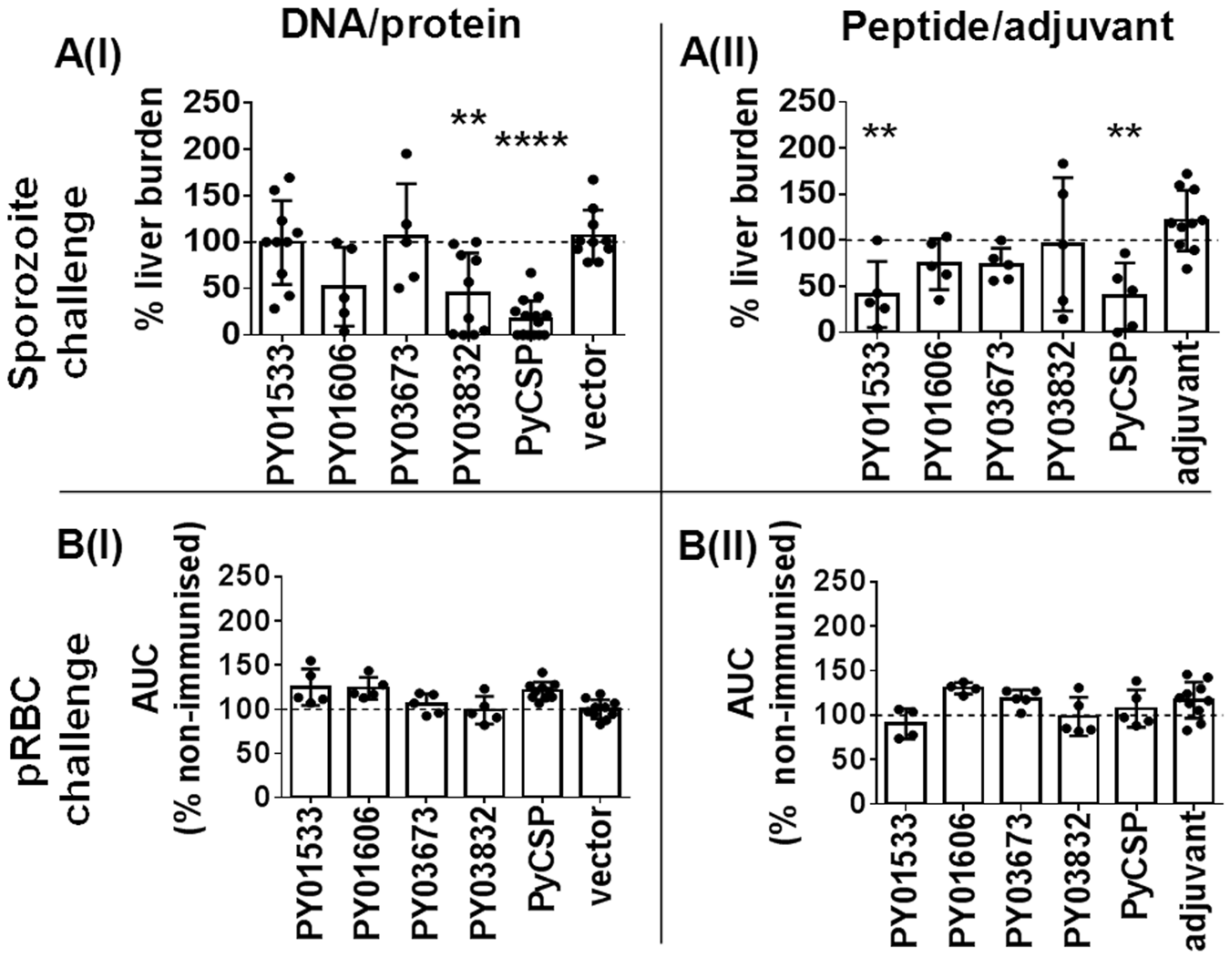
Protective capacity of individual antigens against sporozoite challenge. Mice (n = 5–15/group representative of up to three independent experiments; see Table 3) were immunised with (I) plasmid DNA/recombinant protein expressing the target antigens or (II) pools of synthetic peptides representing predicted CD8^+^ and CD4^+^ T cell epitopes for each antigen formulated in AbISCO100 adjuvant. Controls were immunised with empty vector only (I), or adjuvant only (II), or not immunised **(dotted line)**. Mice were challenged with 10^3^ *P. yoelii* cryopreserved infectious sporozoites or 10^3^ *P. yoelii* pRBC at 14 days post last immunisation. Protection was assessed by **(A)** qRT-PCR of Py18S rRNA in total liver RNA at 42 h after sporozoite challenge normalised against the 18S rRNA of infected but not vaccinated mice (dotted line); and **(B)** FCAB assay for days 3 to 30 after blood-stage challenge normalised against AUC of infectivity controls **(dotted line)**. Data are presented as a scatter plot, with the line representing the mean and the error bars representing standard deviation (SD). Statistical comparison of immunised versus non-immunised infectivity controls was performed using one-way ANOVA followed by Bonferroni’s posthoc test, ****p < 0.0001, ***p < 0.001, **p < 0.01, *p < 0.05. The dotted line represents the mean value for infected but non-immunised control mice used to normalise vaccinated groups and for statistical analysis.

A significant reduction in liver-stage parasite burden following sporozoite challenge was induced by two of the four target antigens: PY03832, 86.4% reduction (*p* = 0.0052) in 60% DNA/protein immunised mice; and PY01533, 73.5% reduction (*p* = 0.0044) in 80% of peptide/adjuvant immunised mice, relative to vector-only or adjuvant controls (Fig. 1A, Table 3). PY01606 (both regimens) and PY03673 (peptide/adjuvant only) also reduced liver-stage parasite burden (37.5–77.4% reduction in 40–60% of mice) but this was not significant relative to controls. Immunisation with PyCSP (positive control) resulted in a significant reduction in liver stage parasite burden following sporozoite challenge (Fig. 1A, Table 3).

Since PY03832, PY01533, and PY01606 (but not PY03673) are expressed in the blood-stage of the parasite lifecycle (Table 1), we further evaluated the capacity of the target antigens to protect against blood-stage parasite challenge. No protection was induced by any of the novel antigens or PyCSP (Fig. 1B).

### Antigen combinations

Protein microarray data^22^ as well as vaccine studies in humans and animal models^34^ suggest that protection against the *Plasmodium* parasite is likely associated with a cumulative response to the signature antigens. Therefore, in addition to assessing the effect conferred by each antigen individually, we also evaluated the immunogenicity and protective capacity of different combinations of our target antigens. The two most protective antigens in our individual antigen studies (PY03832 and PY01533) were combined as ‘Com1/4’, while the other two antigens (PY01606 and PY03673) were combined as ‘Com2/3’; and all four antigens were combined as ‘Com1/2/3/4’.

Impressively, sterile protection was induced by DNA/protein immunisation with Com1/4 in 60% of mice, and Com2/3 in 30% of mice (Table 3); and by peptide/adjuvant immunisation with Com1/4 or Com1/2/3/4 in 20% of mice (Table 3).

**Figure 2:**
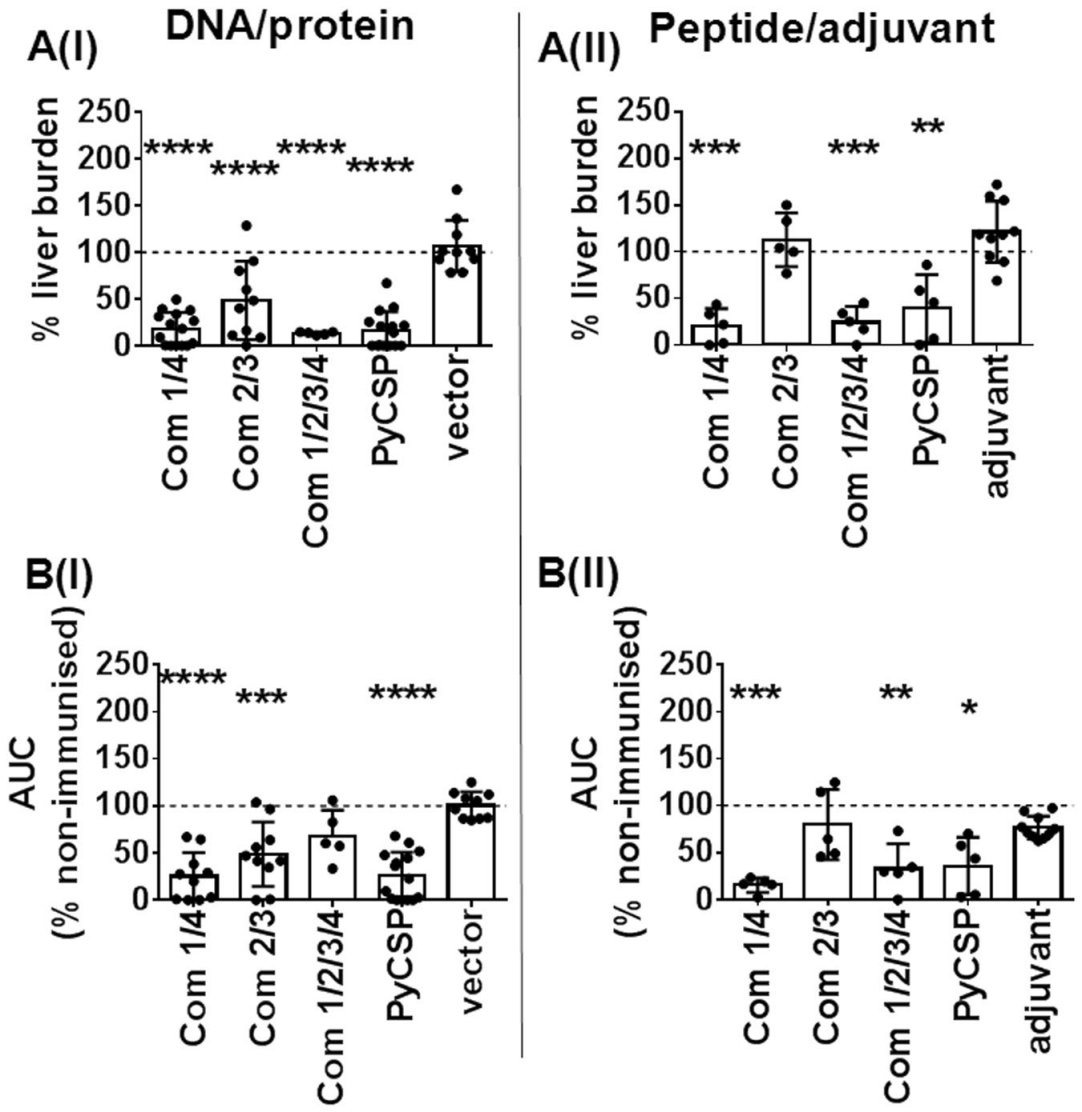
Protective capacity of antigen combinations against sporozoite challenge. Mice were immunized, challenged and protection assessed as described in the legend to Fig. 1. (**A**) liver-stage parasite burden. (**B**) pRBC FCAB. Com1/4 = PY01533 + PY03832; Com2/3 = PY01606 + PY03673; Com1/2/3/4 = PY01533 + PY03832 + PY01606 + PY03673. Data are presented as a scatter plot, with the line representing the mean and the error bars representing standard deviation (SD). Statistical comparison of immunised versus non-immunised infectivity controls was performed using one-way ANOVA followed by Bonferroni’s posthoc test, ****p < 0.0001, *** *p* < 0.001, **p < 0.01, *p < 0.05. The dotted line represents the mean value for infected but non-immunised control mice used to normalise vaccinated groups and for statistical analysis.

A significant reduction in liver-stage parasite burden following sporozoite challenge was induced by both DNA/protein and peptide/adjuvant immunisation with Com1/4 or Com1/2/3/4 (80–82% reduction in 100% mice, *p* ≤ 0.0004; and 76–87% in 100% mice, *p* ≤ 0.0007; respectively); and by immunisation with Com2/3 with the DNA/protein (74% reduction in 70% mice, *p* ≤ 0.0001) but not the peptide/adjuvant regimen (Fig. 2A; Table 3).

In independent experiments, a significant reduction of blood-stage parasite burden following sporozoite challenge was induced with all three combinations in 80–100% of immunised mice: Com1/4 DNA/protein or peptide/adjuvant, 75–84.5% reduction in 100% of mice, *p* ≤ 0.0001; Com2/3 DNA/protein, 64.3% reduction in 80% of mice, *p* = 0.0002; and Com1/2/3/4 peptide/adjuvant, 67% reduction in 100% of mice, *p* = 0.0003 (Table 3; Fig. 2B).

In summary, we established that the *P. yoelii* orthologues of all four *P. falciparum* antigens identified by genome-wide antibody profiling are targets of protection against pre-erythrocytic stage malaria, as evidenced by significant reduction of parasite burden following sporozoite challenge, but no effect on parasitemia following blood stage parasite challenge. Of the four target antigens, PY03832 was the most protective, and PY03673 was the least protective. Notably, the combination of antigens PY03832 and PY01533, termed Com1/4, induced sterile protection in 60% of DNA/protein immunised mice and, in independent experiments, ~80% reduction in liver stage parasite burden and blood-stage parasite burden in 100% of DNA/protein or peptide/adjuvant immunised mice. The combination of antigens PY01606 and PY03673, termed Com2/3, induced sterile protection in 30% of DNA/protein immunised mice and, in independent experiments, 74% reduction in liver stage parasite burden. The combination of all four antigens, Com1/2/3/4, did not further enhance the protective capacity induced by Com1/4 or Com2/3, likely due to the reduced dose of each individual antigen in this combination (see *Methods*).

### Monofunctional analysis of IFN-γ, IL-2 or TNF by CD4^+^ and CD8^+^ T cell responses

Next, we determined whether protection against sporozoite challenge was associated with the induction of Th1 type antigen-specific immune responses. IFN-γ has been implicated with a critical role in protective immunity against malaria and as a potential correlate for protection^35–37^. Thus, we evaluated the IFN-γ response induced by immunisation with our target antigens and antigen combinations via IFN-γ ELIspot and ICS, using splenocytes re-stimulated *in vitro* with A20 APC transfected with plasmid DNA or pulsed with a peptide pool representing the corresponding antigen. The contribution of CD8^+^ versus CD4^+^ T cells was determined by ICS, since CD8^+^ T cells are considered the most important mediator of pre-erythrocytic stage immunity^35,36^. In a first analysis we focused on the production of each cytokine individually by CD8^+^ or CD4^+^ T cell populations.

**Figure 3:**
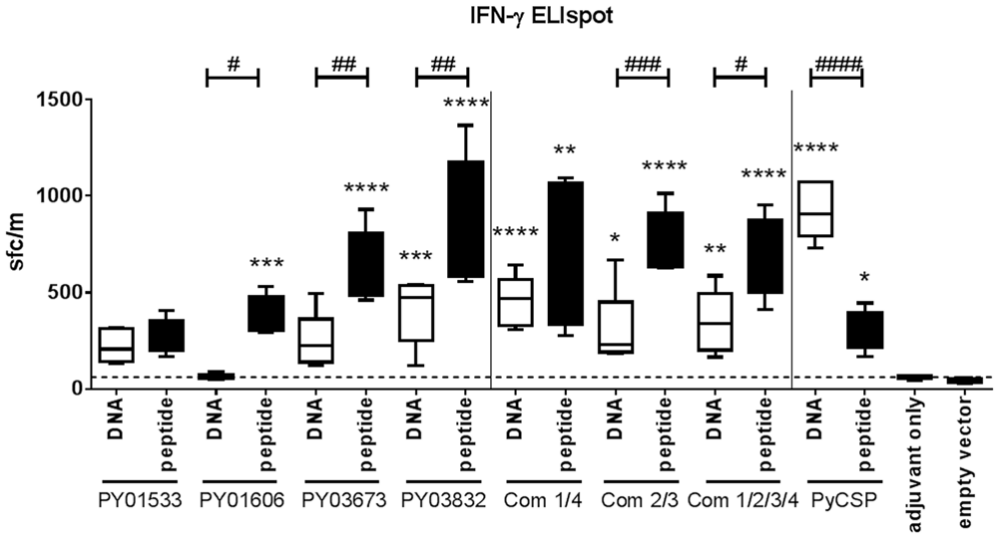
Antigen-specific IFN-γ secreting T cell responses induced by immunisation. Splenocytes were harvested at 14 days post last immunisation and re-stimulated for 48 h with A20 antigen presenting cells transfected with antigen-encoding plasmid DNA or incubated with synthetic peptide pools representing each antigen. Com1/4 = PY01533 + PY03832; Com2/3 = PY01606 + PY03673; Com1/2/3/4 = PY01533 + PY03832 + PY01606 + PY03673. Data are presented as a box and whiskers plot, with the line representing the median and the whiskers illustrating the range for IFN-γ spot forming cells/million splenocytes (n = 5–15/group representative of up to three independent experiments) for (**white bars**) DNA/protein immunisation or (**black bars**) peptide/adjuvant immunisation. Statistical comparison of immunised versus controls (either adjuvant only or empty vector only, for peptide/adjuvant or DNA/protein respectively) was performed using one-way ANOVA followed by Bonferroni’s posthoc test, ****p < 0.0001, ***p < 0.001, **p < 0.01, *p < 0.05. Statistical significance between DNA/protein and peptide/adjuvant immunisation regimens was determined by one-way ANOVA followed by Bonferroni’s posthoc test, ^####^ *p* < 0.0001, ^###^ *p* < 0.0005, ^##^ *p* < 0.005, ^#^ *p* < 0.05. The dotted line represents the mean background value of IFN-γ spot forming cells induced in adjuvant only and empty vector control groups.

In IFN-γ ELIspot, restimulated responses were generally higher using peptide/adjuvant immunisation than the DNA/protein regimen (Fig. 3), thereby validating the epitope prediction algorithms for the identification of peptide-targets of cellular immune mechanisms. The most protective antigen, PY03832, induced robust IFN-γ ELIspot responses that were significantly greater than controls when administered in either peptide/adjuvant or DNA/protein regimens (*p* < 0.0001) (Fig. 3). However, for the other three antigens, there was no correlation of protection with the number of IFN-γ spot forming cells. Consistent with the higher protective efficacy seen with antigen combinations compared to individual antigens, Com1/4, Com2/3 and Com1/2/3/4, all induced highly significant IFN-γ ELIspot responses with both immunisation regimens (*p* < 0.0001–0.05).

**Figure 4:**
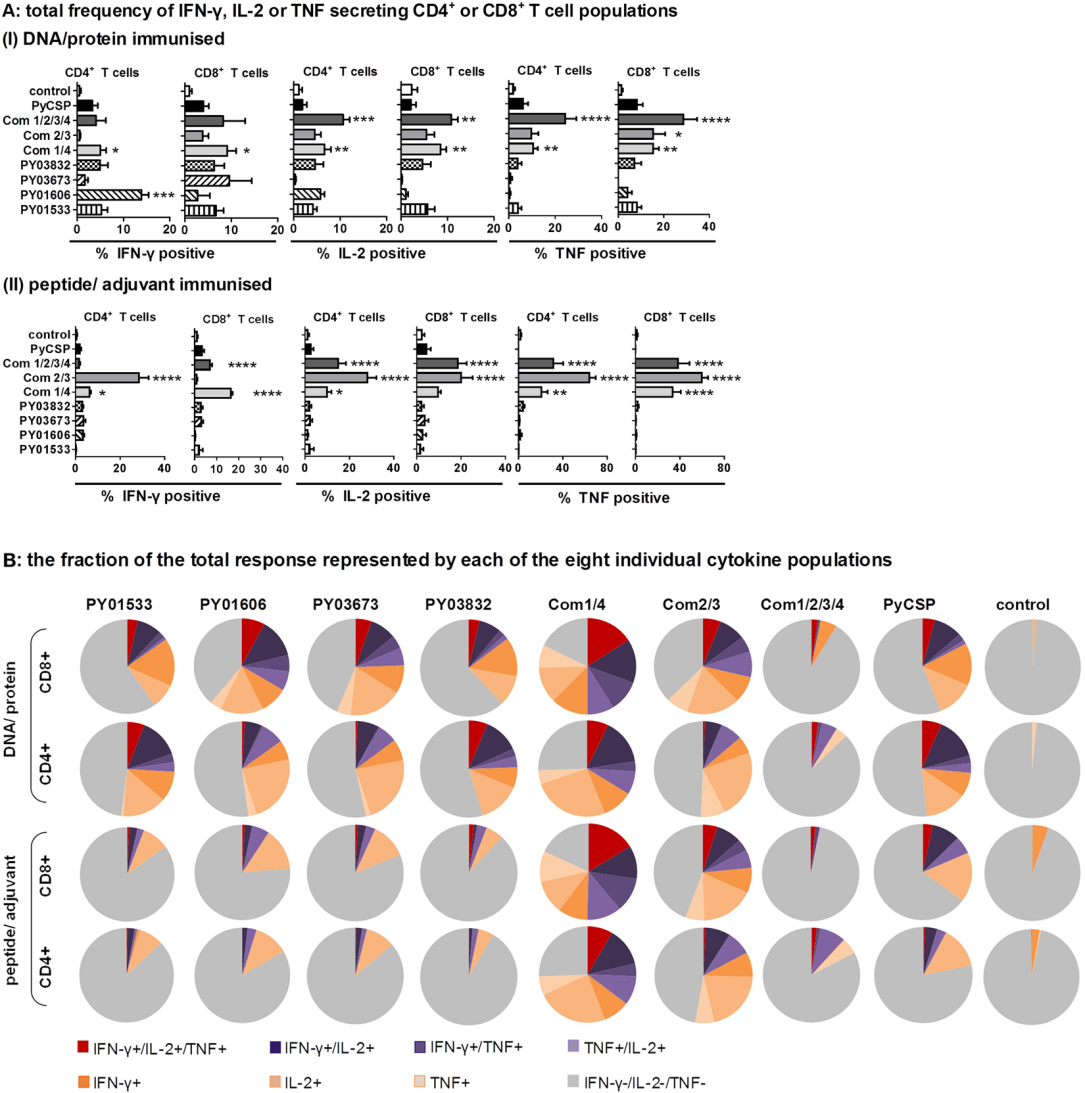
Antigen-specific and combination-specific Th1 cytokine and multifunctional T cell responses. Splenocytes were harvested at 14 days post last immunisation and re-stimulated for 6 h with A20 antigen presenting cells transfected with antigen-encoding plasmid DNA or incubated with synthetic peptide pools representing predicted CD8^+^ and CD4^+^ T cell epitopes for each antigen. Com1/4 = PY01533 + PY03832; Com2/3 = PY01606 + PY03673; Com1/2/3/4 = PY01533 + PY03832 + PY01606 + PY03673. Control = empty vector only or adjuvant only for DNA/protein or peptide/adjuvant groups respectively. Multi-parameter flow cytometry was used to quantify (**A**) total frequency of IFN-γ, IL-2 or TNF secreting CD4^+^ or CD8^+^ T cell populations analysed for each cytokine individually (i.e., total IFN-γ = cells producing only IFN-γ, or IFN-γ + IL-2, or IFN-γ + IL-2 + TNF; etc.) after (I) DNA/protein immunisation or (II) peptide/adjuvant immunisation. **(B)** Fraction of the total response represented by each of the eight possible combinations of IFN-γ, IL-2, or TNF cytokine secreting populations (triple positive, red; double positive, purple; single positive, orange; cytokine negative, grey). Data were analysed using FACS-DiVa software and the FlowJo population comparison tool and are presented as mean frequency (n = 5–15 mice pooled from up to 3 independent experiments) for each population after correction for background cytokine production in mock-stimulated cells. Error bars represent standard deviation (SD). Statistical comparison of immunised versus controls was performed using one-way ANOVA followed by Bonferroni’s posthoc test, ****p < 0.0001, ***p < 0.001, **p < 0.01, *p < 0.05.

For all antigens and combinations, the ICS readout was less sensitive than ELIspot for detecting antigen-induced IFN-γ responses (Figs 3 and 4A). Nonetheless, ICS enabled the analysis of other core Th1 cytokines (IL-2 and TNF) and the phenotype of cytokine secreting cells. All three antigen combinations induced robust IL-2 and TNF responses, as well as IFN-γ responses, with levels exceeding those induced by the individual antigens (Fig. 4A). The effect of antigen combinations appeared to be additive when administered in a DNA/protein regimen and synergistic when administered in a peptide/adjuvant regimen (Fig. 4A). Notably, for Com1/4, there was a significant bias towards expression of Th1 cytokine responses by CD8^+^ T cells rather than CD4^+^ T cells (*p* < 0.0001) with 16.4% of the CD8^+^ T cells secreting IFN-γ and 33.4% of the CD8^+^ T cells secreting TNF following Com1/4 peptide/adjuvant immunisation (Fig. 4A). In contrast, for Com2/3, peptide-induced IFN-γ and IL-2 were secreted almost exclusively by CD4^+^ T cells (each ~28% of CD4^+^ T cells), whereas TNF was secreted by both CD4^+^ and CD8^+^ T cells (59.8–63.8%) (Fig. 4A). For Com1/2/3/4, robust IL-2 and TNF responses of similar magnitude were contributed equally by CD8^+^ and CD4^+^ T cells (IL-2, 15–18.5%; TNF, 31.5–38.3%; p ≤ 0.0001–0.0012)), whereas IFN-γ was significant only for the peptide/adjuvant regimen and the CD8^+^ T cell population (*p* < 0.0001) (Fig. 4A).

Taken together, there was no clear correlation between the frequency of immunisation-induced total Th1 cytokine responses (including total IFN-γ responses) and protection.

### Multifunctional T cell responses

T cells secreting more than one cytokine simultaneously (multifunctional T cells) may provide optimal effector function and have higher capacity to develop into memory cells than single-cytokine secreting cells^38^. Other studies have correlated multifunctional T cells with cell-mediated protection against several pathogens^38–42^, including malaria^43–45^ although this is controversial^46,47^. Therefore, we next determined whether protection following antigen immunisation was associated with the induction of IFN-γ/IL-2/TNF multifunctional T cell populations, by analysing the ICS data using a Boolean gating strategy.

Individual antigens induced robust triple, double and single positive responses when administered in a DNA/protein regimen (Supplementary Figure S1); but, when administered in a peptide/adjuvant regimen, antigen restimulated IFN-γ, IL-2 or TNF T cell populations represented only a small fraction (3–10%) of the total T cell response for all four target antigens (Fig. 4B). The two individual antigens with the highest protective effect on liver-stage parasite burden, PY03832 and PY01533, both induced significant IFN-γ/IL-2/TNF triple positive CD4^+^ as well as CD8^+^ T cell responses (all *p* < 0.0001) (Fig. 4B, Supplementary Figure S1). Conversely, PY01606 and PY03673 both induced significant triple positive CD8^+^ but not CD4^+^ T cell responses (*p* < 0.0001 and *p* = 0.0165, respectively) (Fig. 4B, Supplementary Figure S1). All four antigens individually induced significantly higher frequencies of IFN-γ/IL-2 double positive CD8^+^ or CD4^+^ T cells (all *p* < 0.0001), as well as IFN-γ/TNF CD8^+^ T cells (PY03832 and PY01533, *p* ≤ 0.002; PY01606 and PY03673, *p* < 0.0001) compared to controls (Supplementary Figure S1). Specifically, IFN-γ/TNF CD4^+^ T cells were induced in significant frequencies by the Com1/4 antigens PY0383 and PY01533 (both *p* < 0.0001); while TNF/IL-2 CD8^+^ T cells were induced in significant frequencies only by the Com2/3 antigens PY01606 and PY03673 (both *p* < 0.0001) (Supplementary Figure S1).

Consistent with the increase in protective efficacy, the Com1/4 combination induced very robust and highly significant IFN-γ/IL-2/TNF triple positive CD8^+^ and CD4^+^ T cell responses in both regimens, which were highly synergistic compared to those of the individual antigens (Fig. 4B, Supplementary Figure S1). In contrast, Com2/3 induced CD4^+^ and CD8^+^ T cell responses were similar to those of the individual antigens, with only modest CD8^+^ triple positive responses in either regimen (DNA: *p* = 0.0027; peptide: *p* = 0.0020) and additive effects only noted for IL-2 single positive CD4^+^ T cell populations (Supplementary Figure S1). All three combinations induced significant double positive CD4^+^ and CD8^+^ T cell responses with both regimens: Com1/4 and Com2/3, all *p* < 0.0001; Com1/2/3/4, all *p* ≤ 0.01 (Supplementary Figure S1). In marked contrast, Com1/2/3/4 showed a decrease in the magnitude and variety of multifunctional Th1 cytokine responses, with the triple negative fraction constituting ~85–95% of recall T cell responses (Fig. 4B, Supplementary Figure S1).

Notably, these data fail to show a clear correlation between the frequency of vaccine-induced multifunctional cytokine secreting CD8^+^ or CD4^+^ T cells and protection.

### Association of protection with Th1 types cytokine expression in multifunctional T cell populations

Given the apparent lack of association between protection and frequency of individual cytokine responses or multifunctional CD4^+^ or CD8^+^ T cell populations as reported, we next explored alternative readouts. First, we focused on the median fluorescence intensity (MFI), where the MFI for each fluorescently labelled cytokine expressed by a specific cell population correlates to the amount of cytokine expressed by that specific cell population. In general, the measured MFI for each of the three Th1 cytokines IFN-γ, IL-2 and TNF was higher in triple and double positive T cells than in single positive T cells, but the MFI did not correlate with protection (data not shown).

**Figure 5:**
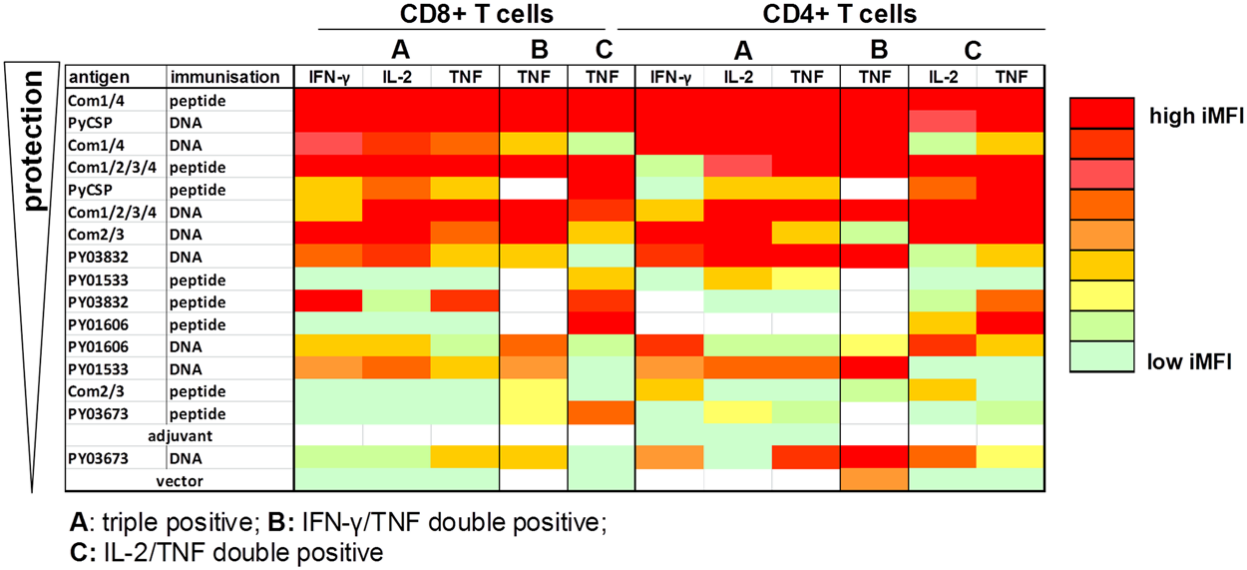
Association of vaccine-induced protective capacity and Th1 cytokine production. The protective capacity of each test group was calculated by determining the mean of the reduction of parasite burden in the liver and in the blood in reference to non-immunised infectivity controls as presented in Table 3, Fig. 1 panel A. The integrated median fluorescence intensity (iMFI) for each multifunctional CD8^+^ or CD4^+^ T cell population (IFN-γ, IL-2 and TNF triple positive, double positive, single positive) was calculated by multiplying the frequency of cells producing a given cytokine combination by the magnitude of the cytokine response (measured as median fluorescence intensity, MFI). Correlation was determined by linear regression analysis using non-parametric Spearman analysis. Data of significantly correlated populations (*p* < 0.05; see Table 4) are ranked according to the protective capacity (see protective index in Table 3) (top: highest protection, bottom: lowest protection).

Next, we evaluated the integrated mean fluorescence intensity (iMFI). The iMFI is computed by multiplying the relative frequency (percent positive) of cells expressing a particular cytokine with the MFI of that population. Accordingly, for both immunisation strategies, individual antigens and antigen combinations (as well as PyCSP control) were ranked according to protective efficacy, by reference to a protective index calculated based on reduction of liver-stage parasite burden and blood-stage parasitemia following sporozoite challenge (see *Methods*) (Supplementary Figure S2). Linear regression analysis (Spearman analysis) revealed a very high correlation between protection and the level of Th1 cytokine expression by triple positive CD8^+^ and CD4^+^ T cells as reflected by the integrated MFI for each cytokine (CD8^+^ T cells: IFN-γ: r = 0.7238, *p* = 0.0007; IL-2: r = 0.8147, *p* < 0.0001; TNF: r = 0.7128, *p* = 0.0009; CD4^+^ T cells: IFN-γ: r = 0.4876, *p* = 0.0401; IL-2: r = 0.8043, *p* < 0.0001; TNF: r = 0.7211, *p* = 0.0007) (Fig. 5, Table 4). Furthermore, expression of high levels of IL-2 by IL-2/TNF double positive CD4^+^ T cell populations (r = 0.5607, *p* = 0.0155) and high levels of TNF by IFN-γ/TNF double positive CD8^+^ (r = 0.5468, *p* = 0.0189) and CD4^+^ (r = 0.5510, *p* = 0.0178) T cell populations, and by IL-2/TNF double positive CD8^+^ (r = 0.6901, *p* = 0.0015) and CD4^+^ (r = 0.7727, *p* = 0.0002) T cell populations were also significantly correlated with protection (Fig. 5, Table 4A).

**Table 4:** Spearman correlation analysis.

A	CD8				CD4			
IFN-γ	+++	++−	+−+	+−−	+++	++−	+−+	+−−
Spearman r	0.7238	0.3934	0.4200	0.2118	0.4876	0.0816	0.2669	-0.1 059
P value (two-tailed)	0.0007	0.1063	0.0827	0.3988	0.0401	0.7476	0.2844	0.6759
**IL-2**	**+++**	**++−**	**−++**	**−+−**	**+++**	**++−**	**−++**	**−+−**
Spearman r	0.8147	0.3955	0.3913	0.0444	0.8043	0.4471	0.5607	0.0898
P value (two-tailed)	<0.0001	0.1043	0.1083	0.8611	<0.0001	0.0629	0.0155	0.7230
**TNF**	**+++**	**+−+**	**−++**	**−−+**	**+++**	**+−+**	**−++**	**−−+**
Spearman r	0.7128	0.5468	0.6901	0.4142	0.7211	0.5510	0.7727	0.4536
P value (two-tailed)	0.0009	0.0189	0.0015	0.0875	0.0007	0.0178	0.0002	0.0587
**B**	**CD8**				**CD4**			
**IFN-γ**	**+++**	**++−**	**+−+**	**+−−**	**+++**	**++−**	**+−+**	**+−−**
Spearman r	0.5625	0.3042	0.3319	0.1947	0.4295	-0.0150	0.2138	-0.1698
P value (two-tailed)	0.0151	0.2197	0.1785	0.4388	0.0753	0.9530	0.3943	0.5006
**IL-2**	**+++**	**++−**	**−++**	**−+−**	**+++**	**++−**	**−++**	**−+−**
Spearman r	0.8125	0.3115	0.2815	0.0005	0.8208	0.3333	0.5439	-0.0418
P value (two-tailed)	<0.0001	0.2083	0.2578	0.9984	<0.0001	0.1765	0.0196	0.8691
**TNF**	**+++**	**+−+**	**−++**	**−−+**	**+++**	**+−+**	**−++**	**−−+**
Spearman r	0.7054	0.5524	0.6362	0.2193	0.7426	0.5673	0.7302	0.3746
P value (two-tailed)	0.0011	0.0174	0.0045	0.3820	0.0004	0.0141	0.0006	0.1257
**C**	**CD8**				**CD4**			
**IFN-γ**	**+++**	**++−**	**+−+**	**+−−**	**+++**	**++−**	**+−+**	**+−−**
Spearman r	0.7408	0.5173	0.2930	0.2909	0.5931	0.4242	0.3120	0.2007
P value (two-tailed)	0.0004	0.0279	0.2380	0.2416	0.0095	0.0793	0.2076	0.4245
**IL-2**	**+++**	**++−**	**−++**	**−+−**	**+++**	**++−**	**−++**	**−+−**
Spearman r	0.6091	0.5264	0.4623	0.2307	0.5773	0.5036	0.5284	0.3435
P value (two-tailed)	0.0073	0.0248	0.0534	0.3570	0.0121	0.0331	0.0242	0.1628
**TNF**	**+++**	**+−+**	**−++**	**−−+**	**+++**	**+−+**	**−++**	**−−+**
Spearman r	0.4099	0.1982	0.3333	0.2969	0.4630	0.2868	0.5010	0.2496
P value (two-tailed)	0.0911	0.4304	0.1765	0.2315	0.0530	0.2485	0.0342	0.3178

+++: IFN-γ/IL-2/TNF triple positive; ++−: IFN-γ/IL-2 double positive; +−+: IFN-γ/TNF double positive; −++: IL-2/TNF double positive; +−−: IFN-γ single positive; −+−: IL-2 single positive; −−+: TNF single positive. A: Protective index = sum of reduction of parasite burden in the liver and in the blood for each immunisation calculated as the mean reduction of parasite burden for all protected mice per group times the percentage of protected mice per group (protection ≥25% reduced parasite burden compared to control group) (see Table 3). B: Protective capacity calculated as mean reduction of parasite burden in the liver for all protected mice times the percentage of protected mice/group (protection ≥25% reduced parasite burden compared to control group). C: Protective capacity calculated as mean reduction of parasite burden in the blood for all protected mice times the percentage of protected mice/group (protection ≥25% reduced parasite burden compared to control group).

The correlation profile with triple positive cytokine expression was very similar when considering protection based only on reduction of parasite burden in the liver (Table 4B), but far less pronounced when considering only protection against blood-stage parasitemia following sporozoite challenge (Table 4C). Here, expression of IFN-γ and IL-2 by IFN-γ/IL-2 double positive CD8^+^ (IFN-γ: r = 0.5173, *p* = 0.0279; IL-2: r = 0.5264, *p* = 0.0248) and CD4^+^ T cell populations (IL-2: r = 0.5036, *p* = 0.0331) was significantly correlated with protection. There was, however, no correlation with expression of high levels of TNF in any double positive populations (both CD8^+^ or CD4^+^) (Table 4C). This is consistent with a role for the liver stage in protection against sporozoite challenge and for TNF early in the induction of protective immunity, while IFN-γ and IL-2 play a role later in the immune response.

### Association of protection with secretion of specific cytokines

We also conducted a more comprehensive analysis of antigen-induced cytokine profiles, assaying the supernatant of splenocytes after *in vitro* re-stimulation for a panel of 10 common cytokines (IFN-γ, IL-2, IL-4, IL-5, IL-6, IL-10, IL-12p70, IL-13, IL1β, TNF) using a cytokine bead array. As for iMFI values, CBA data was ranked according to the protective capacity of each individual antigen or antigen combination for both immunisation regimens.

Consistent with IFN-γ ELISpot and ICS responses, IFN-γ (r = 0.6088; *p* = 0.0073) and TNF (r = 0.5119, *p* = 0.0251) responses were enhanced in protective compared to non-protective groups (Supplementary Figure S3). Furthermore, protection was also significantly correlated with induction of IL-1β (r = 0.4760, *p* = 0.0459) and, especially for the Com2/3 vaccines, IL-6 (r = 0.6034, *p* = 0.0117) (Supplementary Figure S3).

### Parasite-specific antibody responses

Since all four target antigens, PY03832, PY01533, PY01606 and PY03673, were identified in protein microarray studies via parasite-induced antibody reactivity, we anticipated that antibody responses against the parasite would be induced by immunisation with these antigens. Accordingly, we determined the total IgG response against blood-stage parasite extract induced by immunisation with the target antigens in order to evaluate the relative contribution to protection of antibody compared to cellular responses.

At the time of parasite challenge, robust antibody responses relative to controls could be detected only for PY03673 peptide/adjuvant (*p* = 0.0098) (Supplementary Figure S4). Thus, overall, there was no apparent association of antibody responses with protection against sporozoite or blood-stage parasite challenge (Supplementary Figure S4). This is consistent with a prominent involvement of cellular immune responses in the protection induced by immunisation with our novel antigens, rather than humoral responses. We cannot, however, exclude a potential role for antibodies to PY01606 and PY03673 in the partial protection observed after vaccination with these antigens or their combinations, since DNA/protein immunisation with these antigens and both combinations containing these antigens (Com2/3 and Com1/2/3/4) could prime for boosting of antibody responses by parasite challenge (Supplementary Figure S4). There was no apparent priming by antigens PY03832 or PY01533.

### Immunodominant epitopes after immunisation with Com1/4

The robust IFN-γ responses induced in our studies (as reported above) provided the opportunity to identify minimal CD4^+^ and CD8^+^ T cell epitopes derived from the antigens in the most promising antigen combination, Com1/4 (antigens PY01533 and PY03832). Indeed, we identified significant IFN-γ SFC responses to a number of predicted CD8^+^ T cell and CD4^+^ T cell epitopes derived from both PY01533 and PY03832, with a CD8^+^ T cell bias (Supplementary Figure S5).

For PY01533, the IFN-γ response induced by peptide immunisation was broadly distributed with the sum of responses to all of the epitopes (417 SFC/m) approximating that of the pooled peptide responses (626 SFC/m); the most robust responses were directed against two class II restricted peptides (H-2 IA^d^ peptide PY01533 II-3, LMKKCTVKTGVPVQP; IA^d^ percentile 0.65%; 209 SFC/m; and H-2 IE^d^ peptide PY01533 II-9, RRKLLYSLLRCKEGV; IE^d^ percentile 1.62%; 235 SFC7m). In contrast, the IFN-γ response induced by immunisation with PY01533 DNA/protein was directed predominantly against a single CD8^+^ T cell epitope (H-2 D^d^ peptide PY01533 I-14, sequence GDTRLLPIF; D^d^ percentile 0.70%; 205 SFC/m).

For PY03832 a dominance hierarchy was noted within the broad epitope-specific IFN-γ responses induced by peptide immunisation. Here, IFN-γ responses were predominantly directed against two PY03832 peptide epitopes (H-2 IE^d^ peptide PY03832 II-6, sequence YWYKYVYLKKIKKKK; IE^d^ percentile 0.04%; 746 SFC/m; and H-2 IE^d^ peptide PY03832 II-9, CIDDLYWYKYVYLKK; IE^d^ percentile 0.37%; 671 SFC/m); responses to each of these two epitopes were of a similar magnitude to that of the complete peptide pool (626 SFC/m) following peptide/adjuvant immunisation (Supplementary Figure S5). These two peptides share a 10-mer peptide core (YWYKYVYLKK) indicating that this 10-mer peptide may represent a minimal epitope targeted by the antigen-specific IFN-γ responses; indeed, although not selected within the top 1% threshold for study here, this peptide was predicted to bind with high affinity to class I MHC with a H-2 K^b^ score of 6.5.

Another epitope derived from PY03832 (H-2K^d^ peptide PY03832 I-8: VYLKNTNFL; K^d^ percentile 0.33%; 199 SFC/m) was recognised following DNA/protein immunisation, with a 4-fold higher number of IFN-γ SFC compared to controls and a 2.6-fold higher number of IFN-γ SFC compared to the complete peptide pool, suggesting that the entire IFN-γ response induced by protein immunisation was directed against this epitope (Supplementary Figure S5).

## Discussion

It is widely considered that a highly effective malaria vaccine will need to include multiple antigens, likely from multiple stages of the complex *Plasmodium* parasite life cycle, including novel antigens identified from the parasite genomic sequence^17,48^. Herein, we evaluated four previously uncharacterized *P. falciparum* proteins identified from antibody-based genome-wide screening platforms as potential pre-erythrocytic malaria vaccines candidates. Three of these proteins (*P. falciparum* PFL1620 = *P. yoelii* PY03832; MALP1.22 = PY01533; PF10925w = PY01606) were identified in the signature of 16 *P. falciparum* antigens associated with protective immunity induced by experimental immunisation with *P. falciparum* radiation attenuated sporozoites (RAS) as well as in the signature of 46 *P. falciparum* antigens associated with naturally acquired immunity)^19,22^. The fourth antigen (PF14_0051 = PY03673) ranked very high in the RAS signature list and was moderately reactive with antibodies in various naturally exposed populations in other protein microarray studies^22^. Our studies establish that all four proteins identified via antibody-based screening are targets of cell mediated immune responses and protection targeting the pre-erythrocytic stage of the *Plasmodium* parasite. In particular, a 74–86% reduction in liver-stage parasite burden following sporozoite challenge was induced in 60–80% of mice immunised with antigens PY03832 and PY01533, and the combination of those antigens (Com1/4) resulted in a synergistic increase in the protective capacity in both DNA/protein and peptide/adjuvant formulations with sterile protection in 60% of immunised mice. A synergistic effect on protection was also observed upon immunisation with the combination of PY01606 and PY03673 (Com2/3) when administered as DNA/protein but not peptide/adjuvant. These data establish each of our four target antigens as promising candidates for malaria vaccine development.

The increase in protective capacity of multi-antigen combinations confirms our previous findings from protein microarray studies suggesting that cumulative reactivity against multiple antigens is more strongly associated with protection than reactivity to individual antigens^22^. This reinforces the concept that multiplicity of parasite targets would overcome genetic restriction, low immunological responsiveness and parasite evasion of the immune response^48^. Studies in rodent challenge models have established proof-of-concept for this, with protection demonstrated following bivalent immunisation with PyCSP plus PyHEP17 that was not induced with the individual antigens^34^ and subsequently with combinations of recently identified antigens (PY03424, PY0311, PY03661^49^; or recently identified antigens (SLAPR/SAP1 or LISP1) when combined with CSP^50^; or a previously identified antigen (TRAP) when combined with CSP^51^. Another study showed that viral vectors expressing a newly identified *P. falciparum* antigen PfLSP2 (as well as a previously identified vaccine candidate antigen PfLSA1) induced sterile protection against transgenic *P. berghei* sporozoite challenge in mice which was dependent on CD8^+^ T cells^52^.

A growing body of data now exists in humans on multi-antigen combinations of blood-stage antigens^53,54^ as well as antigens expressed across several stages of the parasite lifecycle^55,56^. Importantly, one study in malaria-naïve humans showed an unprecedented level of sterile immunity (27%; 4/15 subjects) against *P. falciparum* sporozoite challenge following immunization with a combination of adenovirus-based subunit vaccines encoding CSP and AMA1^57^; protection was associated with cellular immune responses. A more recent study combining recombinant adenovirus/poxvirus expressing TRAP with the CSP-based RTS,S/AS01B protein vaccine resulted in minimal enhancement of efficacy with 82.4% (14/17) of malaria-naive adults protected by immunization with adenovirus/poxvirus TRAP plus RTS,S/versus 75% (12/16) immunized with RTS,S/AS01B alone^58^ emphasizing the importance of both antigen and delivery method.

Findings of synergy with DNA vectors encoding PY03832 and PY01533, and PY01606 and PY03673, show that correct antigen selection is important in antigen combinations. The protective capacity of Com1/4 and Com2/3 formulations in reducing blood stage parasite burden was not further increased when all four antigens were combined (Com1/2/3/4), even though this combination showed a high reduction in liver stage parasite burden. Notably, Com1/2/3/4 induced lower frequencies of multi-functional T cell responses compared to more protective formulations, which further highlights the complexity of cellular immune responses required for protection. The observed reduced protective capacity and cellular immune response of Com1/2/3/4 may be due to the reduced dose of each antigen in the four-antigen combination, consistent with a dose-dependent protective effect, or may be explained by antigenic competition, with antigen-derived peptide epitopes competing for the same binding site on MHC molecules^59^. The potential issue of antigenic competition with multi-component DNA, poxvirus adenovirus and/or protein based malaria vaccines has been reported previously in murine and nonhuman primate studies, showing both inhibition and lack of inhibition of antigen-specific immune responses or protective efficacy^60–66^. Thus, immune interference associated with multi-antigen mixtures is complex and may be antigen-dependent^67^.

Our earlier protein microarray studies showed a lack of correlation between the magnitude of antibody response (serodominance) and protection, suggesting that antigens recognised by antibodies and associated with protection are likely to be also recognised by T cells^22^. We here confirm that antigens identified in a serological screen are targets of cellular immunity. All four target antigens induced robust CD4^+^ and CD8^+^ T cell responses. The two most promising vaccine antigens, PY01533 and PY03832, induced high levels of IFN-γ, IL-2 and TNF expressing CD4^+^ and CD8^+^ T cells, as well as IFN-γ/IL-2/TNF triple positive CD4^+^ and IFN-γ/TNF double positive CD4^+^ T cell populations. The increased protective capacity of the combination of these two antigens (Com1/4) compared to single antigen vaccination was accompanied by a synergistic increase in IFN-γ/IL-2/TNF triple positive CD8^+^ T cell populations expressing higher levels of IFN-γ, IL-2 and TNF than the individual antigens alone. Interestingly, with Com1/4 immunisation, secretion of Th1 type cytokines was strongly biased towards CD8^+^ T cells whereas the less protective combination Com2/3 had a CD4^+^ T cell bias. Moreover, although the level of pre-erythrocytic stage protection induced by immunisation with Com1/4 was not markedly higher than that induced by the leading malaria vaccine target CSP, Com1/4 induced a higher frequency of IFN-γ expressing CD8^+^ and CD4^+^ T cells, as well as triple positive and double positive cytokine secreting T cells, whereas CSP induced a much more robust sporozoite-targeted antibody response. Considering their predicted function, our novel pre-erythrocyte antigens are unlikely to be expressed on the parasite surface or secreted by sporozoites, but might be presented on the surface of infected hepatocytes in the context of MHC molecules. Thus, we propose that antibodies targeting sporozoite entry are not involved in mediating protection, but antibodies targeting late liver-stage parasites or blood-stage parasites might contribute to the observed reduction in blood-stage parasitemia. Notably, there was only very limited antibody reactivity before or after parasite challenge with blood-stage parasite extract, suggesting that the observed reduction in blood-stage parasitemia was due to reduced liver-stage parasite burden. This suggests a different mechanisms of protection compared to CSP, and is consistent with clinical studies of the CSP-based malaria vaccine RTS,S/AS01, where the predominant mechanism of protection is considered to be sporozoite-targeted antibodies and not antigen-specific CD8^+^ T cells^68^. Vaccination with RTS,S induces high levels of antibodies and CD4^+^ T cells specific for CSP; and protection against malaria has been associated with antibodies especially to the repeat region in RTS,S^69,70^ with a significant synergistic interaction identified between CSP-specific CD4^+^ T cells and anti-CSP antibodies in determining protection against clinical malaria^71^. There is no evidence that RTS,S induces CSP-reactive CD8^+^ T cell responses^71–73^.

The findings of our study support the increasing consensus among immunologists that simple measurements of the magnitude of immune responses are insufficient to predict immune protection. Rather phenotypical characterisation of T cell responses associated with multiple effector cytokines may provide key information with regard to the immune control^74,75^. The induction and maintenance of multifunctional, virus-specific CD8^+^ T cells has been associated with control of virus infections including EBV, CMV, influenza, and non-progressive HIV-1^76–78^. Furthermore, studies in mice have shown that vaccine-induced multifunctional CD4^+^ T cell populations which produce more cytokine on a per-cell basis than monofunctional cells, and thus have more potent effector capability, correlate with protection against *Leishmania major* challenge^38^. Herein, we demonstrate that protection against *Plasmodium* sporozoite challenge is highly correlated with the induction of multifunctional T cell response. Other studies in rodent and non-human primate models of malaria have associated IFN-γ production in CD8^+^ T cells, but not multifunctional T cell responses, with protection^46,47^. However, our data are consistent with a report showing a significant increase in the proportion of CD62L^−^ CD45RO^+^ effector memory T cells producing multiple cytokines (IFN-γ, TNF and IL-2), but not individual cytokines, in humans protected against sporozoite challenge by experimental infection with *P. falciparum* sporozoites under chloroquine chemoprophylaxis, at time of challenge as compared with baseline^43^. Also, in a phase 2a RTS,S/AS malaria vaccine study in malaria-naïve volunteers, there was a strong association between the titers of CSP-specific antibodies and the frequency of CSP peptide-reactive CD4^+^ T cells in the same individual, and the frequencies of CD4^+^ T cells secreting either or both IL-2 and TNF-α (but not IFN-γ) were higher in protected than non-protected subjects^44^.

In summary, in this report we describe the first validation for any pathogen of novel antigens identified in protein microarray studies as targets of cellular immune responses and sterilising infection-blocking protection. In particular, antigens PY01533 and PY03832 and that two-antigen combination (Com1/4) proved highly protective when administered in either DNA/protein or peptide/adjuvant regimens. Importantly, this is the first report correlating protection against virulent *Plasmodium* parasite challenge with high-level cytokine production by multifunctional T cell populations. Our results provide experimental validation for the concept of rational vaccine design from genomic sequence data, demonstrate that antigens identified by serological screening are targets of cellular immune responses, and correlate protection against pre-erythrocytic stage malaria with multifunctional Th1 cytokine secreting cells and not monofunctional T cells.

## Methods

### Mice and parasites

Specific pathogen-free female BALB/c mice (Animal Resource Centre, Perth, Australia) were immunised at 6 weeks of age. The parasite strain used in all experiments was *Plasmodium yoelii* 17XNL. Cryopreserved sporozoites kindly provided by Dr. Stephen Hoffman (Sanaria Inc., Rockville, MD, USA) were used for sporozoite challenge. Parasitised red blood cells (pRBC) used for blood-stage challenge were obtained after one passage of frozen pRBC stock (derived from cryopreserved sporozoites at 14 days post infection) through a BALB/c mouse. All studies were approved by the QIMR Animal Ethics Committee and were conducted in accordance with the Australian Code of Practice for the Care and Use of Animals for Scientific Purposes (2004).

### Parasite extract

*P. yoelii* extract was generated from *P. yoelii* 17XNL grown in BALB/c mice to approximately 50% parasitemia, as previously described^79^. Briefly, blood was collected by cardiac puncture, diluted with 10 volumes of FCAB buffer (1x PBS, 0.5% FCS, 2 mM EDTA), and centrifuged at 600 rcf for 10 min. The cell pellet was resuspended in 0.5% w/v saponin (Sigma-Aldrich, Castle Hill, NSW) in FCAB buffer and incubated for 30 min at 37 °C. The sample was then twice drawn through a 30-gauge needle, washed with 20 volumes of Milli-Q H_2_0 and centrifuged as above. pRBC were fixed in FCAB fixation and lysis buffer (1x PBS, 4% w/v paraformaldehyde and 0.0067% w/v saponin) for 10 min at 37 °C. The extract was resuspended in FCAB buffer and stored at −80 °C prior to use. All buffers were sterile filtered (0.2 µm) immediately before use.

### Synthetic peptides

Putative CD4^+^ and CD8^+^ T cell epitopes were predicted from the translated full-length genes of the *P. yoelii* orthologues of the four target proteins (PY01533, PY01606, PY03673, PY03832) (Table 1) and PyCSP (www.PlasmoDB.org ^80,81^ using computerised MHC-binding prediction algorithms available from the Immune Epitope Data Base (www.iedb.org)^82^. Peptides were synthesised by Mimotopes Pty Ltd (Clayton, VIC) at a purity of >80% and resuspended in 100% dimethyl sulfoxide (DMSO) and stored at −80 °C prior to use. For each antigen, a pool of all putative CD8^+^ and CD4^+^ T cell epitopes was used to immunise mice.

### Generation of plasmid vectors for DNA vaccination and recombinant protein production expression

*P. yoelii* 17XNL genomic DNA was extracted from pRBC of BALB/c mice 14 days after infection with *P. yoelii* 17XNL sporozoites. Full-length gene sequence of the *P. yoelii* orthologues of each target antigen (PY01533, PY01606) or a partial length sequence corresponding to the protein fragment highly immunoreactive in protein microarray screening platforms (PY03673, PY03832)^22^ (www.PlasmoDB.org) were amplified by PCR. Gene-specific oligonucleotide primers flanked with restriction enzyme sites or sequence overhangs for DNA fusion were designed using Amplify 3.1 (http://engels.genetics.wisc.edu/amplify/) and Oligo Calc (www.basic.northwestern.edu/biotools/oligocalc.html) software tools and were commercially synthesised by Sigma-Aldrich (Castle Hill, NSW) or Geneworks (Hindmarsh, SA).

The antigenic sequence for PY03673 includes exons 2 and 4 that were fused using an enzymatic DNA assembly method as previously described^83^. Briefly, each exon was amplified with a 40 bp overhang complementary to the other exon. The resulting DNA fragments were combined in one reaction with *Exonuclease III* (ExoIII), heat-stable *taq DNA ligase* and Phusion® High Fidelity *DNA polymerase* (all from New England Biolabs, Ipswich, MA) in CBAR buffer (150 mM Tris-HCl pH7.5, 10 mM MgCl2, 10 mM DTT, 800 µM dNTPs, 1 mM NAD, 5% PEG-800). The enzymatic assembly reaction was incubated at 37 °C for 5 min for optimal 3′ exonuclease activity, then at 72 °C for 20 min to stop digestion of DNA by ExoIII; then the temperature was reduced by 0.1 °C/second to 60 °C and then held at 60 °C for 1 h (optimal temperature for DNA polymerase and DNA ligase) to complete ligation and DNA repair.

PY03832 is a 10 kb long gene, so the *P. yoelii* sequence corresponding to the antigenic fragment of PFL1620w, exon 1 segment 3^22^ was selected to represent PY03832 for our DNA/protein immunisation studies (peptide epitopes were predicted from the full-length sequence). Gene sequences of *P. falciparum* PFL1620w and its *P. yoelii* PY03832 orthologues were aligned using ClustalW2 (www.ebi.ac.uk/Tools/msa/clustalw2/) and BLASTnt (blast.ncbi.nlm.nih.gov/Blast.sgi) and the genetic *P. yoelii* fragment corresponding to PFL1620w exon 1 segment 3 was identified.

Amplification of each target gene from *P. yoelii* genomic DNA was conducted in a 50-μl PCR reaction containing 1 unit/μl KOD Hot Start *DNA polymerase* in corresponding buffer (Novagen, San Diego, CA), 0.4 μM dNTPs, 333 nM of each primer and 100 ng DNA template. PCR conditions were: initial denaturation at 95 °C for 2 min; 30 cycles at 95 °C for 30 sec, 50 °C for 45 sec and 68 °C for 1 min/kb; and a final extension at 68 °C for 10 min. The PCR products were purified using the QIAquick PCR purification kit (Qiagen, Germany), and digested overnight at 37 °C using the restrictions enzymes whose recognition site was inserted into the primer sequences.

Genes were then cloned into pVR1020 (Vical, Inc., San Diego, California)^84^ using standard methods. The resulting plasmid was amplified in *E. coli* DH5α for DNA vaccination and purified using an endotoxin-free plasmid extraction kit (EndoFree Plasmid Giga Kit, Qiagen Pty Ltd, Chadstone Centre, VIC).

For protein expression, a custom pIVEX-HIS/HA vector was created by modifying the pIVEX-HIS 3.2 vector (Roche Applied Science, Castle Hill, NSW) to express a C-terminal HA tag in addition to the N-terminal HIS tag^85^. The cell-free protein expression system RTS 500 HY *E. coli* (5 Prime GmbH, Hamburg, Germany) was used to produce recombinant protein, according to manufacturer’s instructions. Recombinantly expressed proteins were HIS purified using Cobalt Talon Resin (Clontech Laboratories, Inc., Mountain View, CA). Briefly, Talon resin was equilibrated with wash buffer (20 mM sodium phosphate, 500 mM NaCl, 10 mM Imidazole) and incubated for 30 min at 4 °C with the RTS product solution in wash buffer containing 8 M urea. The mixture was then transferred to an Econo-Pac chromatography column (Bio-Rad Laboratories Pty. Ltd., Gladesville, NSW), the endotoxin was removed with wash buffer containing 0.05% Triton X-114, and the purified protein eluted with 150 mM Imidazole. To exchange the elution buffer with PBS, the protein eluate was loaded onto a 4 ml 3 K Amicon Ultra Filtration tube (Merck Millipore, Billerica, MA) and concentrated in three alternating spinning and washing steps. The protein concentration after the final centrifugation step was measured using a Nanodrop Spectrometer (Thermo Fisher Scientific) with an A260/A280nm ratio below 1.8 considered indicative of pure protein. The endotoxin level of purified protein products was assessed by endpoint chromogenic LAL assay (QCL-1000, Lonza Group, Ltd., Basel, Switzerland) according to manufacturer’s instructions and ranged between 0.5–0.9 EU/ml. Protein quality after cell-free expression was also evaluated by SDS-PAGE and western blot, using the N-terminal HIS tag to detect recombinant protein. As expected, full-length as well as partial length protein of varying sizes was produced in the cell-free system (Supplementary Figure S6), covering all possible epitopes and effectively boosting DNA primed responses.

### Immunisation and Challenge

BALB/c mice (n = 5/group) were immunised in independent experiments at three weekly intervals with either individual antigens or antigen combinations, in both DNA prime/protein boost and peptide/adjuvant immunisation regimens. For the DNA/protein regimen, mice were immunised with two intramuscular doses (tibialis anterior muscle; dose split between two legs) of 100 µg plasmid DNA followed by one intraperitoneal dose of 10 µg recombinant protein formulated 50:50 in Alhydrogel® (Alum; Brenntag Biosector, Denmark). For the peptide/adjuvant regimen, mice were immunised with three subcutaneous doses of pooled synthetic peptides (29.5 µg/peptide, 10% DMSO) formulated with 12 µg AbISCO100 (Isconova, Sweden). The same total dose of DNA, protein or peptide was used for immunisations with individual antigens and the antigen combinations, so the amount of individual antigens in combinations was reduced accordingly. Mice administered empty pVR1020 prime followed by a 50:50 Alum/1x PBS boost were used as the control group for DNA/protein studies (vector control), and mice administered AbISCO100 adjuvant only were used as the control group for peptide/adjuvant studies (adjuvant control).

Two weeks after the last immunisation, mice were challenged with either 1000 cryopreserved sporozoites in 1% mouse sera or 1000 pRBC in PBS, in a volume of 200 µl intravenously in the tail vein. The sporozoite dose was based on counts conducted before freezing; we routinely use cryopreserved sporozoite stocks for *in vivo* challenge experiments^32,33,79^ and have established that 100% of mice inoculated with as few as 50 cryopreserved *P. yoelii* sporozoites developed blood-stage parasitemia^79^. Infectivity controls (non-immunised naïve mice challenged with parasites in parallel to the test groups) were included in all experiments, and all developed blood-stage parasitemia from day 4 post sporozoite challenge (data not presented). The well characterized antigen, *P. yoelii* circumsporozoite protein (PyCSP) administered in parallel to the test groups served as a positive control for all immunogenicity and protection studies.

### Assessment of liver-stage parasite burden via quantitative RT-PCR

Livers were harvested 42 h after sporozoite challenge and liver-stage parasite burden quantitated by qRT-PCR as previously described^32,79^. *P. yoelii* 18 S rRNA (Py18S) and mouse GAPDH were quantified using a standard curve of cloned cDNA and the ratio of Py18S cDNA to total mouse cDNA (GAPDH) was calculated and normalised against the infectivity control group to give the percent reduction of liver parasite burden (Microsoft Excel 2007, version 12, Microsoft Corporation, WA, USA).

### Assessment of blood-stage parasitemia via flow cytometric assessment of blood (FCAB assay)

The kinetics and burden of blood-stage infection were monitored daily from day 3 to day 14 and 3 times a week from day 14 to day 30 after either sporozoite or pRBC challenge, by flow cytometric assessment of the blood (FCAB) as described previously^32,33^. The percentage of fluorescent cells was measured by flow-cytometry on an HTS-equipped FACSCanto II (BD Biosciences, San Jose, CA) and data were analysed using FlowJo software version 9.1 (Treestar Inc., Ashland, OR, USA). The percentage of pRBCs was measured at multiple time points over the course of infection and the Area Under the Curve (AUC) of blood-stage parasitemia over time was calculated for immunised and control groups. In order to compare different experiments, AUC values of immunised mice were normalised to AUC values of infectivity control mice.

A value expressing the protective capacity for each antigen/vaccine combination was calculated for immunised mice and control groups by multiplying the average reduction of parasite burden in the liver (% determined by qRT-PCR) and the average reduction of blood-stage parasitemia (% determined by FCAB) with the number of protected mice (protection defined as >25% reduction of parasite burden compared to infectivity controls) and calculating the sum of liver-stage and blood-stage protective index:

*(average reduction of liver-stage parasite burden in protected mice [%]* × *number of protected mice/group)* + *(average reduction of blood-stage parasitemia [%]* × *number of protected mice/group)*.

### Immunogenicity assays

Spleens of vaccinated and unvaccinated mice were harvested at the time of challenge (12 days after the third immunisation) and re-stimulated with A20 (mouse B lymphocyte cell line, ATCC # TIB-208) antigen presenting cells transfected with the corresponding plasmid DNA or pulsed with the corresponding peptide pool. Briefly, A20 cells were transfected with antigen-encoding plasmid DNA (5 µg DNA/5 × 10^6^ cells) using the AMAXA Nucleofector system (kit V, program L-13; Lonza Group, Ltd., Basel, Switzerland) according to manufacturer’s instructions, or pre-incubated with 5 µg/ml peptide pool, and then irradiated with approximately 16666 cGy (Cs137 irradiator). Then, 5 × 10^5^ splenocytes/well were co-incubated with 1.5 × 10^5^ antigen-presenting A20 cells/well at 37 °C for 6–48 hr depending on the assay.

### IFN-γ ELISpot assay

IFN-γ secretion was assayed by ELISpot as previously described^79^, using 5 × 10^5^ splenocytes/well and 1.5 × 10^5^ peptide-pulsed or antigen-transfected A20 APCs/well in triplicate (for individual mice) and a 42 hr incubation period. Spot forming cells (SFCs) were counted using the AID ELISpot reader (AID ELISpot reader classic, AID, San Diego, CA).

### Cytometric Bead Array (CBA)

A comprehensive panel of Th1 and Th2 cytokines (IL-1β, IL-2, IL-4, IL-5, IL-6, IL-10, IL-12, IL-13, IFN-γ, TNF) was assayed using the mouse cytometric bead array (CBA) flex kit (BD Biosciences, San Jose, CA) also as previously described^32^, with 5 × 10^5^ splenocytes/well and 1.5 × 10^5^ peptide-pulsed or antigen-transfected A20 APCs/well in 96-well round-bottom plates (for individual mice) and a 48 hr incubation period. Fluorescence intensity of the detection beads was measured on the FACSArray (BD Biosciences, San Jose, CA) and data analysed using FlowJo software (FlowJo software version 9.1 Treestar Inc.).

### Intracellular Cytokine Staining (ICS)

The phenotype and frequency of cytokine secreting cells and multifunctional T cell populations was determined by Intracellular Cytokine Staining (ICS), as previously described^79^, with 5 × 10^5^ splenocytes/well and 1.5 × 10^5^ peptide-pulsed or antigen-transfected A20 APCs/well in 96-well round-bottom plates and a 6 hr incubation period. Briefly, cells were stained for surface molecules CD4 (mCD4-PerCP Cy5, Invitrogen) and CD8 (mCD8-FITC, Biolegend), fixed with 1% paraformaldehyde, and then stained for intracellular cytokines IFN-γ (mIFN-γ-APC, Miltenyi Biotec), IL-2 (mIL-2-PE, Biolegend), and TNF (mTNF-PE Cy7, eBioscience) in 1x Perm/Wash^TM^ buffer (BD Bioscience) overnight at 4 °C in the dark. Samples were acquired on an HTS-equipped FACS Canto II using FACS-DiVa software (BD Bioscience).

The percentage of IFN-γ, IL-2 or TNF positive CD4^+^ and CD8^+^ splenocyte populations was calculated by subtracting background values of mock-stimulated cells from the antigen-specific re-stimulated sample using Overton subtraction in the FlowJo Population Comparison tool (FlowJo software version 9.1 Treestar Inc.).

CD4^+^ and CD8^+^ T cell populations expressing one, any two, or all three cytokines simultaneously (multifunctional) were defined using the FlowJo Boolean Gating tool (FlowJo software version 9.1 Treestar Inc.). The background frequencies of cytokine producing populations in mock-stimulated samples were subtracted and statistical analysis of data was performed using Prism version 6.00. The median fluorescence intensity (MFI) of the three cytokines in single, double or triple positive T cell populations relates to the amount of cytokine secreted from each of these populations. The integrated MFI (iMFI) was therefore calculated by multiplying the MFI for each individual cytokine with the relative frequency of multifunctional populations expressing that cytokine, as an indicator of T cell functionality.

### Indirect fluorescent antibody test (IFAT)

Sera of vaccinated and unvaccinated mice were collected 2 weeks after each immunisation (after the third immunisation sera were collected 4 days before challenge) and 21 days after challenge. Parasite specific antibody responses were assayed using a flow-based Indirect fluorescent antibody test (IFAT) as previously described^79,86^ using 30 µl of serum diluted in FCAB buffer (1/100 pre-challenge, 1/300 post-challenge) and incubated with 10 µl of parasite extract for 20 min at RT. Antibodies binding to the *P. yoelii* parasite extract were stained with secondary goat anti-mouse IgG-DyLight 405 (Biolegend) for 15 min at 4 °C. Samples were acquired on a HTS-equipped FACSCanto II and post-acquisition data analysis was performed with FlowJo (software version 9.1 Treestar Inc.). The MFI for total IgG measured in immunised mice was normalised to that of infectivity controls.

### Statistical Analysis

The Prism 6.0 software package (Graph-Pad, San Diego, CA) was used for statistical analysis. For each test group, all mice were assayed individually and geometric mean responses were calculated. Results obtained after vaccination with DNA/protein formulations of PY01533, PY03832, combination 1/4 and combination 2/3 were confirmed in independent experiments, so the number of samples per group included in the data analyses varied: PyCSP and Com1/4 DNA/protein, n = 15; PY01533, PY03832 and Com2/3 DNA/protein, n = 10; PY01606, PY03673 and Com1/2/3/4 DNA/protein, n = 5; Peptide/adjuvant studies, n = 5. One-way ANOVA followed by Bonferroni’s correction for multiple comparisons was used to determine statistical significance between immunised and control groups. Correlation and linear regression analysis of protection with multifunctional T cell data was conducted using the non-parametrical Spearman analysis. Correlation and linear regression analysis of protection with iMFI and CBA data was conducted using the non-parametrical Spearman analysis (GraphPad Prism). Error bars in all figures represent standard deviation (SD).

### Data Availability

The authors declare that the data supporting the findings of this study are available within the article and its Supplementary Information files.

## Electronic supplementary material

Supplementary Information
