# Supplementary material for: Novel Plasmodium antigens identified via genome-based antibody screen induce protection associated with polyfunctional T cell responses
Source: Sci Rep. 2017 Nov 8;7:15053. doi: 10.1038/s41598-017-15354-0 (PMC5678182; doi:10.1038/s41598-017-15354-0)
Supplement: Supplementary file 1 — Supplementary Information [file 41598_2017_15354_MOESM1_ESM.pdf]

SUPPLEMENTARY INFORMATION:

Sophie Schussek, Angela Trieu, Simon H Apte, John Sidney, Alessandro Sette, and Denise L Doolan

Novel Plasmodium antigens identified via genome-based antibody screen induce protection associated with polyfunctional T cell responses

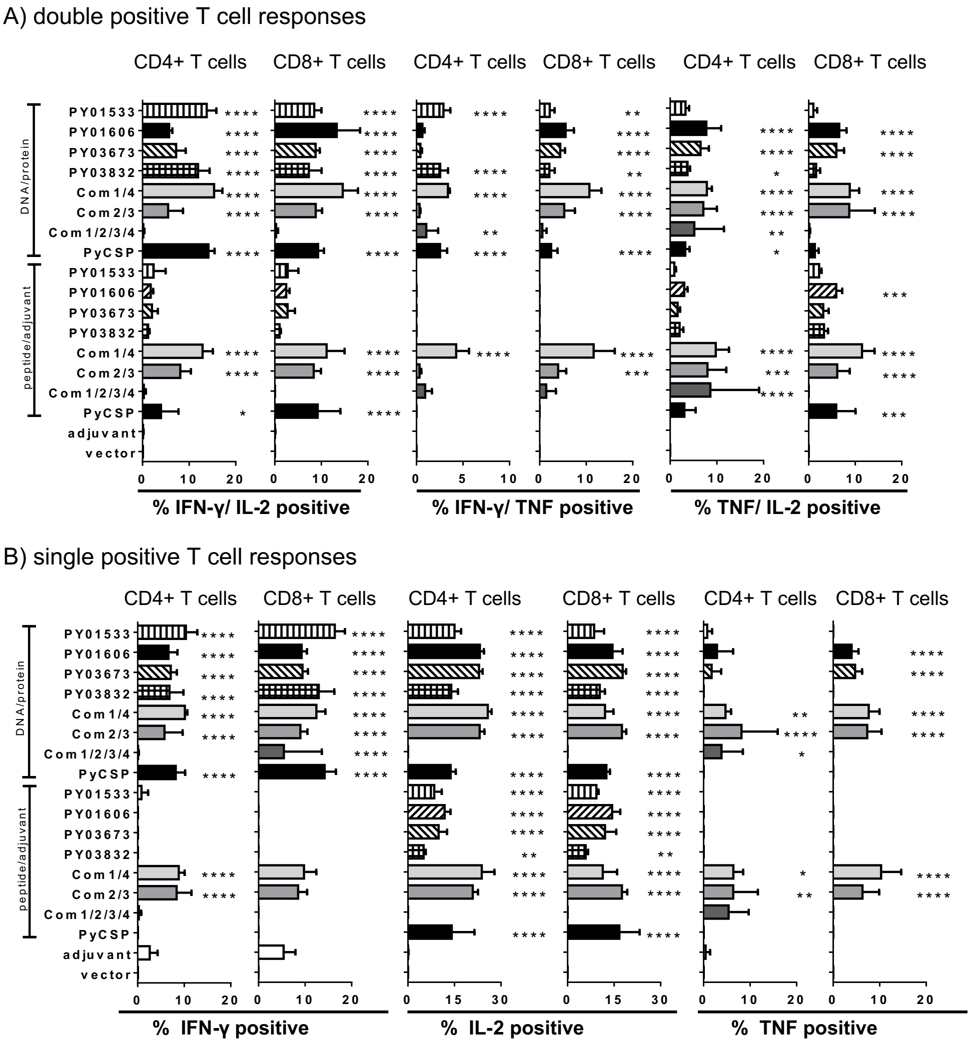

Supplementary Figure S1: Double and single positive T cell populations. Splenocytes were harvested at 14 days post last immunisation and re-stimulated for 6h with A20 antigen presenting cells transfected with antigen-encoding plasmid DNA or incubated with synthetic peptide pools representing predicted $CD8^+$ and $CD4^+$ T cell epitopes for each antigen. Com1/4 = PY01533+PY03832; Com2/3 = PY01606+PY03673; Com1/2/3/4 = PY01533+PY03832+PY01606+PY03673. Multi-parameter flow cytometry was used to quantify **(A)** frequency of $CD4^+$ or $CD8^+$ T cell populations expressing any two of the three cytokines, IFN-γ, IL-2 or TNF **(B)** frequency of $CD4^+$ or $CD8^+$ T cell populations expressing only one of the cytokines IFN-γ, IL-2, and TNF, respectively. Error bars represent standard deviation (SD). Statistical comparison of immunised versus controls (empty vector or adjuvant only, for DNA/protein or peptide/adjuvant groups respectively) was performed using one-way ANOVA followed by Bonferroni's posthoc test, **** p<0.0001, *** p<0.001, ** p<0.01, * p<0.05.

+++ IFN-γ/ IL-2/TNF triple positive
++- IFN-γ/ IL-2 double positive
+-+ IFN-γ/ TNF double positive
-++ IL-2/TNF double positive
+-- IFN-γ/ single positive
-+- IL-2 single positive
--+ TNF single positive
--- triple negative

high iMFI

low iMFI

I) IFN-γ iMFI

protection

antigen	immunisation strategy	CD8								CD4							
		+++	++-	+-+	-++	+--	-+-	--+	---	+++	++-	+-+	-++	+--	-+-	--+	---
Com1/4	DNA	908.2	782.4	111.3	n.d.	791.2	n.d.	n.d.	n.d.	726.5	1130.7	154.7	n.d.	304.4	n.d.	n.d.	n.d.
Com1/4	peptide	1781.0	765.5	1234.3	n.d.	683.2	n.d.	n.d.	n.d.	1357.0	1382.1	581.0	n.d.	946.8	n.d.	n.d.	n.d.
Com1/2/3/4	peptide	1232.2	79.5	379.1	7.8	294.1	0.2	3.2	n.d.	173.9	14.7	62.3	n.d.	15.2	n.d.	n.d.	n.d.
CSP	DNA	1377.1	584.0	233.1	n.d.	557.0	n.d.	n.d.	n.d.	1051.0	949.1	288.5	n.d.	291.8	n.d.	n.d.	n.d.
Com2/3	DNA	1958.2	373.8	464.1	n.d.	320.9	n.d.	n.d.	n.d.	1646.2	3972.8	249.4	16.8	3997.3	n.d.	12.5	n.d.
Com1/2/3/4	DNA	542.2	65.0	114.1	3.1	649.2	0.3	0.5	n.d.	316.7	48.6	122.2	n.d.	79.2	n.d.	n.d.	n.d.
CSP	peptide	642.5	1685.6	n.d.	194.2	n.d.	307.3	25.0	n.d.	129.4	761.5	n.d.	94.1	n.d.	n.d.	96.6	n.d.
PY03832	DNA	779.1	815.0	108.8	0.7	868.8	n.d.	n.d.	n.d.	659.8	1053.7	160.3	n.d.	483.1	n.d.	n.d.	n.d.
PY01606	DNA	619.7	1231.3	364.7	1.8	467.8	n.d.	n.d.	n.d.	766.9	3482.0	541.7	n.d.	4091.0	n.d.	n.d.	n.d.
PY01533	peptide	156.0	447.3	n.d.	39.0	n.d.	20.1	46.5	n.d.	127.1	492.0	n.d.	7.9	129.0	101.5	n.d.	n.d.
PY03832	peptide	1899.8	136.7	n.d.	43.1	n.d.	33.8	n.d.	n.d.	n.d.	247.0	n.d.	20.4	n.d.	31.1	n.d.	n.d.
PY01606	peptide	127.8	332.3	n.d.	111.0	n.d.	110.5	n.d.	n.d.	n.d.	355.2	n.d.	26.0	n.d.	43.0	n.d.	n.d.
Com2/3	peptide	192.4	426.9	130.7	0.4	430.3	n.d.	n.d.	n.d.	373.3	6391.9	163.6	n.d.	6502.8	n.d.	n.d.	n.d.
PY03673	peptide	145.3	383.6	n.d.	54.3	n.d.	108.1	n.d.	n.d.	103.9	394.7	n.d.	40.6	n.d.	95.7	n.d.	n.d.
PY01533	DNA	700.6	704.6	150.6	2.8	892.2	5.8	2.2	n.d.	451.3	781.3	195.5	7.2	473.8	2.9	4.6	69.2
PY03673	DNA	294.6	374.6	221.1	n.d.	380.6	n.d.	n.d.	n.d.	497.5	4740.9	303.3	n.d.	4624.9	n.d.	n.d.	n.d.
empty vector		194.2	n.d.	170.6	35.9	n.d.	n.d.	n.d.	n.d.	n.d.	n.d.	75.4	4.1	n.d.	n.d.	n.d.	142.1
adjuvant only		n.d.	4.3	n.d.	n.d.	373.4	n.d.	n.d.	n.d.	3.4	16.3	n.d.	n.d.	223.5	n.d.	n.d.	n.d.

II) IL-2 iMFI

protection

antigen	immunisation strategy	CD8								CD4							
		+++	++-	+-+	-++	+--	-+-	--+	---	+++	++-	+-+	-++	+--	-+-	--+	---
Com1/4	DNA	788.1	507.5	0.8	129.1	n.d.	546.5	n.d.	n.d.	443.8	636.5	n.d.	213.4	n.d.	561.0	n.d.	n.d.
Com1/4	peptide	1113.2	491.3	n.d.	611.0	n.d.	407.6	n.d.	n.d.	649.8	627.5	n.d.	547.7	n.d.	968.7	n.d.	n.d.
Com1/2/3/4	peptide	3173.4	135.1	427.9	150.9	n.d.	5.8	34.1	n.d.	276.3	18.5	10.7	1496.0	n.d.	34.5	93.4	n.d.
CSP	DNA	1496.9	443.4	4.2	323.5	2.2	623.1	n.d.	n.d.	715.3	592.9	n.d.	327.7	n.d.	567.0	n.d.	n.d.
Com2/3	DNA	893.5	335.4	0.6	433.0	n.d.	561.4	n.d.	n.d.	412.2	227.3	0.1	1021.8	n.d.	750.8	n.d.	n.d.
Com1/2/3/4	DNA	1600.9	151.7	163.5	89.6	3.3	5.7	7.5	n.d.	517.3	52.7	14.6	1097.0	n.d.	n.d.	31.2	n.d.
CSP	peptide	697.7	1184.4	n.d.	651.6	n.d.	1324.9	n.d.	n.d.	129.3	636.7	n.d.	309.2	n.d.	1186.7	13.6	n.d.
PY03832	DNA	755.2	554.8	0.8	93.1	n.d.	560.7	n.d.	n.d.	423.9	621.6	n.d.	185.3	n.d.	629.7	n.d.	n.d.
PY01606	DNA	493.1	1016.8	n.d.	312.0	n.d.	474.9	n.d.	n.d.	55.6	206.9	3.0	406.3	13.8	801.3	16.5	n.d.
PY01533	peptide	219.3	335.7	n.d.	274.8	n.d.	658.4	n.d.	n.d.	137.2	591.6	n.d.	95.9	n.d.	682.0	n.d.	n.d.
PY03832	peptide	270.6	95.3	n.d.	283.8	n.d.	378.8	n.d.	n.d.	24.4	229.2	n.d.	186.9	n.d.	367.5	n.d.	n.d.
PY01606	peptide	102.5	212.4	n.d.	543.2	n.d.	1034.6	n.d.	n.d.	n.d.	240.7	n.d.	246.1	n.d.	910.5	n.d.	n.d.
Com2/3	peptide	173.9	391.7	n.d.	166.3	n.d.	743.8	n.d.	n.d.	31.1	356.8	n.d.	270.7	n.d.	813.9	n.d.	n.d.
PY03673	peptide	198.9	288.4	n.d.	321.0	n.d.	834.6	n.d.	n.d.	89.1	350.8	n.d.	149.8	n.d.	769.7	n.d.	n.d.
PY01533	DNA	741.8	455.6	1.2	59.0	n.d.	371.3	n.d.	n.d.	266.6	436.5	0.9	157.3	n.d.	523.8	0.2	33.6
PY03673	DNA	277.3	348.4	n.d.	288.9	n.d.	703.4	n.d.	n.d.	31.7	261.1	n.d.	332.3	n.d.	869.5	n.d.	n.d.
empty vector		153.3	n.d.	34.2	99.9	n.d.	n.d.	45.3	n.d.	n.d.	n.d.	14.9	9.5	n.d.	n.d.	23.1	19.0
adjuvant only		n.d.	10.3	n.d.	n.d.	94.8	n.d.	62.5	n.d.	4.1	23.5	n.d.	n.d.	29.2	13.4	36.2	16.3

III) TNF iMFI

protection

antigen	immunisation strategy	CD8								CD4							
		+++	++-	+-+	-++	+--	-+-	--+	---	+++	++-	+-+	-++	+--	-+-	--+	---
Com1/4	DNA	3024.2	229.4	375.5	689.8	94.7	106.6	n.d.	n.d.	2519.3	157.9	563.7	1390.8	3.3	32.5	n.d.	n.d.
Com1/4	peptide	4790.1	7.5	3304.4	2687.3	4.8	0.2	2691.1	n.d.	5036.1	192.1	2194.7	4705.8	73.0	137.7	3015.8	n.d.
Com1/2/3/4	peptide	23505.1	772.0	9540.9	2621.1	n.d.	27.3	751.8	n.d.	3828.3	n.d.	725.9	21043.9	n.d.	n.d.	5826.1	n.d.
CSP	DNA	4795.2	416.1	937.0	1641.3	225.6	408.9	274.7	n.d.	3712.4	344.4	1186.6	2164.6	29.0	129.4	271.8	n.d.
Com2/3	DNA	2755.1	3.9	787.6	1103.1	0.4	1.1	671.5	n.d.	476.5	2.2	53.7	3653.6	n.d.	398.4	1345.9	n.d.
Com1/2/3/4	DNA	22790.2	688.5	3614.3	1402.4	18.7	27.0	176.4	n.d.	3510.9	n.d.	1072.0	7669.5	n.d.	n.d.	4016.4	n.d.
CSP	peptide	1743.6	239.8	n.d.	2396.9	n.d.	1054.3	202.7	n.d.	418.3	689.6	n.d.	2040.3	n.d.	1421.7	729.8	n.d.
PY03832	DNA	2451.6	82.4	427.7	521.6	74.9	110.8	n.d.	n.d.	2274.3	98.9	634.6	1228.8	21.1	32.7	n.d.	n.d.
PY01606	DNA	884.0	n.d.	608.6	658.1	n.d.	n.d.	368.2	n.d.	158.5	n.d.	111.2	1342.2	n.d.	557.4	694.7	n.d.
PY01533	peptide	302.0	115.1	n.d.	989.7	n.d.	70.1	n.d.	n.d.	304.6	295.9	n.d.	676.2	n.d.	371.5	24.8	n.d.
PY03832	peptide	12991.1	82.3	n.d.	1626.6	n.d.	472.7	1355.7	n.d.	64.2	233.5	n.d.	1630.3	n.d.	575.6	2486.8	n.d.
PY01606	peptide	444.5	186.8	n.d.	2731.0	n.d.	828.8	665.0	n.d.	n.d.	338.4	n.d.	2124.6	n.d.	1231.6	1188.4	n.d.
Com2/3	peptide	318.9	n.d.	213.2	294.9	n.d.	n.d.	263.8	n.d.	80.5	n.d.	32.7	829.0	n.d.	241.6	494.0	n.d.
PY03673	peptide	393.2	177.1	n.d.	1401.0	n.d.	376.3	227.0	n.d.	204.3	384.7	n.d.	1121.0	n.d.	797.9	544.1	n.d.
PY01533	DNA	2130.0	56.4	505.6	320.2	n.d.	n.d.	26.0	n.d.	1325.4	n.d.	617.9	847.5	n.d.	0.0	398.5	n.d.
PY03673	DNA	2451.6	82.4	427.7	521.6	74.9	110.8	n.d.	n.d.	2274.3	98.9	634.6	1228.8	21.1	32.7	n.d.	n.d.
empty vector		357.0	n.d.	480.5	468.8	n.d.	n.d.	523.2	n.d.	n.d.	n.d.	279.5	44.7	n.d.	n.d.	379.9	87.4
adjuvant only		n.d.	n.d.	n.d.	n.d.	n.d.	n.d.	n.d.	n.d.	6.6	n.d.	n.d.	n.d.	n.d.	n.d.	n.d.	155.6

Supplementary Figure S2: Association of vaccine-induced protective capacity and $CD4^+$ or $CD8^+$ Th1 cytokine production.

The protective capacity of each test group was calculated by determining the mean of the reduction of parasite burden in the liver and in the blood in reference to no-vaccine controls as presented in Table 3. The integrated median fluorescence intensity (iMFI) for each multifunctional $CD8^+$ or $CD4^+$ T cell population **(I)** IFN-γ iMFI; **(II)** IL-2 iMFI; **(III)** TNF iMFI) was calculated by multiplying the frequency of cells producing a given cytokine combination by the magnitude of the cytokine response (measured as median fluorescence intensity, MFI). Data are ranked according to the protective capacity (see protective index in Table 3) (top: highest protection, bottom: lowest protection).

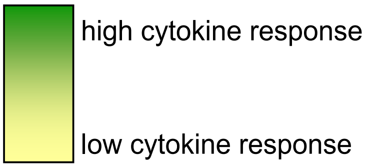

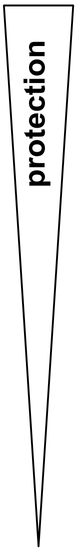

antigen	immunisation	IFN-γ	IL-1β	IL-6	TNF	IL-4	IL-10	IL-12p70	IL-2	IL-13	IL-5
Com1/4	peptide	873.9	6.0	8.5	28.6	1.4	4.0	11.2	1.9	n.d.	1.2
PyCSP	DNA	531.6	22.0	252.5	332.3	1.3	4.7	10.4	4.2	15.4	1.8
Com1/4	DNA	1111.1	33.6	49.4	78.4	7.1	160.6	88.9	10.0	9.4	18.1
Com1/2/3/4	peptide	676.7	n.d.	170.3	115.2	2.2	176.3	n.d.	224.3	433.9	96.1
PyCSP	peptide	17.2	5.6	9.6	14.7	2.3	5.1	17.2	38.5	5.8	1.6
Com1/2/3/4	DNA	301.9	3.1	16.2	53.7	0.3	17.8	30.1	1.5	3.2	0.1
Com2/3	DNA	301.9	10.6	176.9	623.1	0.6	39.6	n.d.	3.5	11.2	n.d.
PY03832	DNA	39.3	0.2	n.d.	51.0	0.4	n.d.	28.9	24.1	77.9	0.5
PY01533	peptide	245.5	2.5	29.7	49.5	0.3	31.6	4.1	1.0	7.8	0.5
PY03832	peptide	36.7	5.2	3.8	38.0	1.1	312.8	2.2	17.1	472.2	31.4
PY01606	peptide	245.5	2.5	29.7	49.5	0.3	31.6	4.1	1.0	7.8	0.5
PY01606	DNA	9.2	18.5	5.3	5.5	1.6	1.4	2.5	10.5	45.2	0.3
PY01533	DNA	410.1	0.4	39.9	15.3	0.5	n.d.	0.4	1.6	4.0	n.d.
Com2/3	peptide	707.1	2.6	133.9	1.2	5.0	196.6	192.2	132.1	908.9	354.2
PY03673	peptide	224.3	0.5	7.4	10.2	n.d.	2.3	5.6	0.8	0.6	0.8
	adjuvant	n.d.	9.8	n.d.	142.8	n.d.	7.4	17.4	n.d.	11.9	1.4
PY03673	DNA	56.7	10.2	1.4	14.1	0.8	5.3	3.8	0.6	59.3	2.6
vector		0.1	0.5	0.6	21.9	n.d.	16.2	n.d.	1.1	n.d.	n.d.
non-immunised		0.3	n.d.	1.4	17.9	0.5	24.1	n.d.	1.8	1.9	1.3

Supplementary Figure S3: Association of vaccine-induced protective capacity and antigen-specific and combination-specific cytokine responses by Cytokine Bead Array analysis.

Splenocytes were harvested at 14 days post last immunisation, re-stimulated for 48h with A20 antigen presenting cells transfected with antigen-encoding plasmid DNA or by incubation with peptide pools representing each antigen, and the amount of secreted cytokines was analysed. The protective capacity was calculated and correlated to cytokine responses as described in the legend to Figure 6. Cytokine data for significantly correlated cytokine species are presented in pg/ml (mean of data from one n=5 or two n=10 independent experiments) after correction for background cytokine production in mock-stimulated cells, and arranged according to the protective capacity (top: highest protection, bottom: lowest protection).

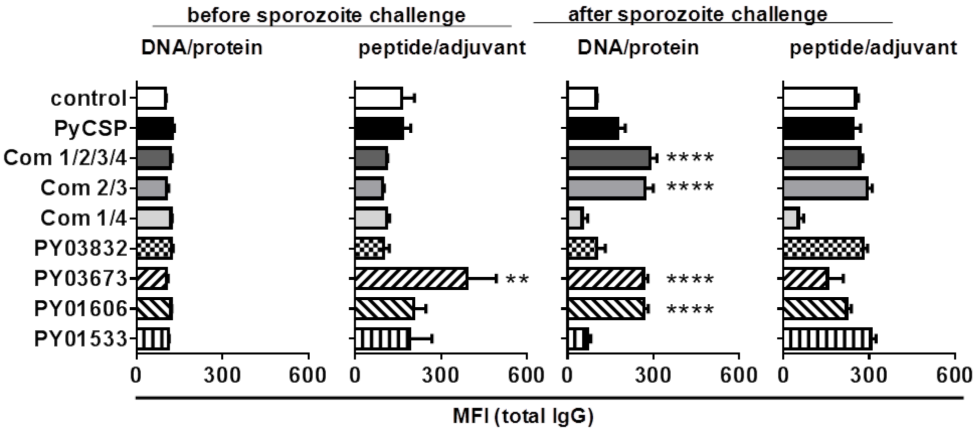

Supplementary Figure S4: Antigen-specific and combination-specific antibody responses.

Flow cytometric analysis of total IgG levels binding to *P. yoelii* blood stage extract in the serum of DNA/protein or peptide/adjuvant immunised mice before or 23 days after sporozoite challenge. Com1/4 = PY01533+PY03832; Com2/3 = PY01606+PY03673; Com1/2/3/4 = PY01533+PY03832+PY01606+PY03673. Control= empty vector only or adjuvant only for DNA/protein or peptide/adjuvant groups respectively. Data are presented as Median Fluorescence Index (MFI) with standard deviation (SD), correlating to the antibody titre, normalised against non-immunised controls (n=5 mice/group). Statistical comparison of immunised versus non-immunised controls was performed using one-way ANOVA followed by Bonferroni's posthoc test, **** $p<0.0001$, *** $p<0.001$, ** $p<0.01$, * $p<0.05$.

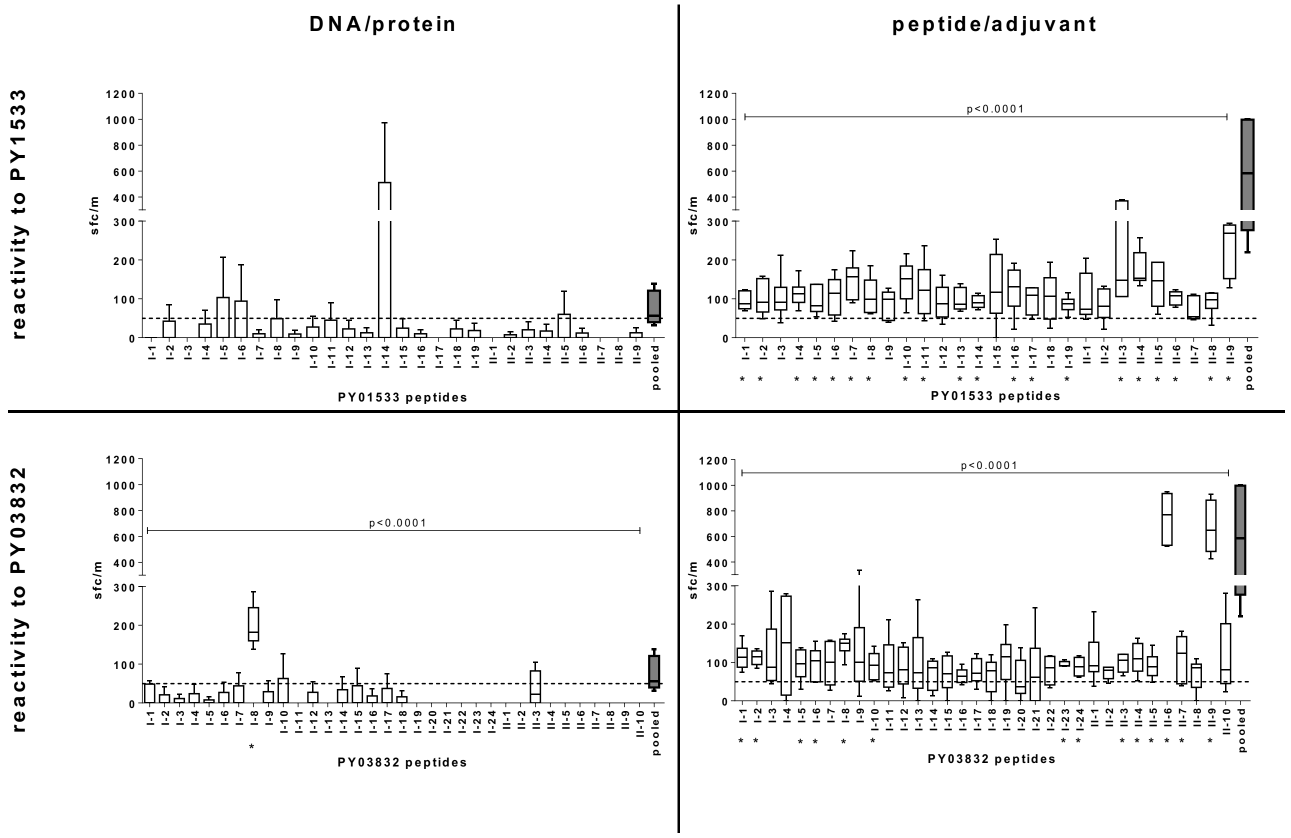

Supplementary Figure S5: Identification of novel T cell epitopes.

IFN-γ production of splenocytes harvested from mice immunised with Com1/4 DNA/protein **(left)** or Com1/4 peptide/adjuvant **(right)** at 14 days post last immunisation and re-stimulation for 48h *in vitro* with individual synthetic peptides representing predicted $CD8^+$ and $CD4^+$ T cell epitopes for antigens PY03832 and PY01533 or the corresponding peptide pool for each antigen. Data are presented as mean spot forming cells (SFCs)/million splenocytes for individual mice (n=5/group) with error bars representing the total range of values. The dotted line represents the mean background value of IFN-γ spot forming cells induced in adjuvant only and empty vector control group. Statistical significance across all peptide stimulations was determined by one-way ANOVA.

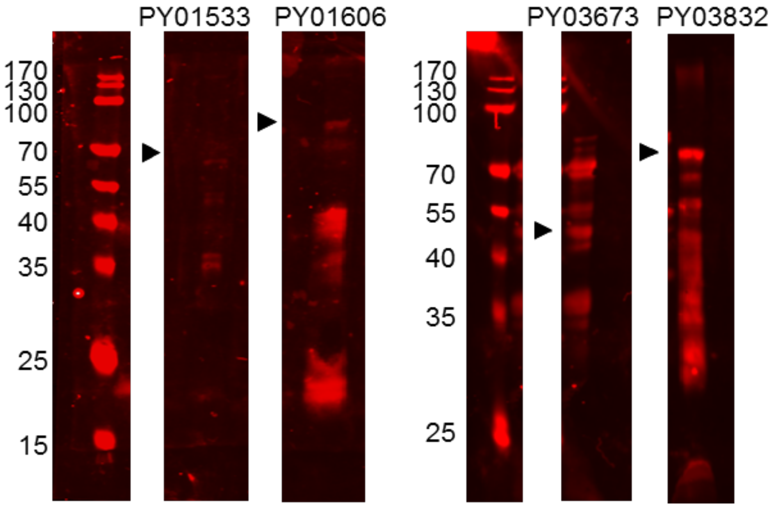

Supplementary Figure S6: SDS-PAGE of recombinant proteins. Proteins were expressed from a custom pIVEX-HIS/HA vector using the cell-free protein expression system RTS 500 HY *E. coli*. Recombinantly expressed proteins were HIS purified using Cobalt Talon Resin and endotoxin was removed with wash buffer containing 0.05% Triton X-114. After elution and buffer exchange with PBS, proteins were loaded onto a 4ml 3K Amicon Ultra Filtration tube (Merck Millipore, Billerica, MA) and concentrated in three alternating spinning and washing steps. Purified samples were separated by 10% SDS-PAGE and HIS-tagged proteins were detected by Western Blot in PVDF-FL membranes using IRDye-680 conjugated anti-HIS antibodies. As expected, full-length as well as partial length protein of varying sizes was produced in the cell-free system, covering all possible epitopes and effectively boosting DNA primed responses.

Supplementary Table 1: Top 1%: MHC class I and top 2% MHC class II binding epitope predicted for each antigen: Predicted for mouse MHC class I alleles H-2K^{d} and H-2D^{d} and for mouse MHC class II alleles IA^{d} and IE^{d} using algorithms available on the Immune Epitope Database (www.IEDB.org).

Antigen	MHC-peptide	Sequence	Length	Position	H-2 Kd percentile	H-2 Dd percentile
PY01533	I-1	SYEPSRRSL	9	640	0.09	49.52
PY01533	I-2	SYYEVSDKL	9	730	0.14	72.69
PY01533	I-3	AYSRTEASI	9	176	0.18	27.79
PY01533	I-4	KYDGERAQI	9	476	0.82	58.97
PY01533	I-5	LSPVHTAAI	9	759	13.88	0.14
PY01533	I-6	SKPNLFFNI	9	366	13.14	0.33
PY01533	I-7	EGDTRLLPI	9	377	35.33	0.40
PY01533	I-8	ELPNIEMII	9	416	50.92	0.41
PY01533	I-9	YGAFVLAAY	9	683	24.02	0.44
PY01533	I-10	IMPGVTESI	9	517	13.17	0.47
PY01533	I-11	YEPSRRSLN	9	641	18.56	0.54
PY01533	I-12	FKDSLLFTF	9	93	50.26	0.55
PY01533	I-13	FDAHYVWEI	9	744	24.35	0.63
PY01533	I-14	GDTRLLPIF	9	378	66.63	0.70
PY01533	I-15	GIGLRFPRF	9	775	85.04	0.74
PY01533	I-16	GVPVIKEPL	9	575	35.20	0.75
PY01533	I-17	GKGKRSGVY	9	675	83.71	0.78
PY01533	I-18	ILPFQILTT	9	540	75.22	0.87
PY01533	I-19	IFPLPRLTI	9	208	10.13	0.95
PY01606	I-1	NYLNGPSSI	9	722	0.13	40.05
PY01606	I-2	NYDTSNNEI	9	801	0.21	35.53
PY01606	I-3	SGLGGVQAI	9	136	0.34	4.32
PY01606	I-4	NYEESDEYI	9	975	0.36	47.12
PY01606	I-5	SSLFVRDPI	9	608	0.37	23.75
PY01606	I-6	IYSYNREFI	9	452	0.41	9.90
PY01606	I-7	QITMSFQTI	9	490	0.50	16.60
PY01606	I-8	GFLKIGTPL	9	2	0.67	10.11
PY01606	I-9	SPIKINKTI	9	187	0.75	50.23
PY01606	I-10	SPHSRYVTL	9	343	0.84	2.05
PY01606	I-11	NGPSSIGWR	9	725	33.35	0.01
PY01606	I-12	CTPYLGGFL	9	521	29.33	0.07
PY01606	I-13	TGASFLRKF	9	867	73.79	0.16
PY01606	I-14	GTPSVPFKF	9	104	90.83	0.17
PY01606	I-15	ISPHSRYVT	9	342	42.33	0.23
PY01606	I-16	PPPILDNNY	9	715	91.00	0.26
PY01606	I-17	SSPINLSPI	9	181	1.68	0.51
PY01606	I-18	WTPEYSSFT	9	93	68.48	0.60
PY01606	I-19	KNPIKNYVY	9	468	97.53	0.80
PY01606	I-20	ELPLKKNYY	9	569	84.80	0.84
PY01606	I-21	VDPHKERIK	9	918	99.00	0.85
PY01606	I-22	TPEYSSFTI	9	94	4.88	0.91
PY01606	I-23	TLPCYPNVF	9	145	78.15	0.94
PY03673	I-1	FYIGISDNI	9	1350	0.04	19.31
PY03673	I-2	FYNHTINSI	9	798	0.06	8.82
PY03673	I-3	KYTNTDSNV	9	1130	0.11	96.77
PY03673	I-4	IYIQGKNIL	9	313	0.24	74.53
PY03673	I-5	CYENRNNFL	9	465	0.26	7.37
PY03673	I-6	SYKLRDDDI	9	61	0.27	18.69
PY03673	I-7	LYFASINRK	9	755	0.28	88.79
PY03673	I-8	VYLKNTNFL	9	346	0.30	19.33
PY03673	I-9	MYVRMDEDI	9	1313	0.43	27.48
PY03673	I-10	SYMIKENNL	9	1250	0.55	25.25
PY03673	I-11	KYYESEIQV	9	1291	0.64	90.09
PY03673	I-12	KYEKQLTEF	9	1409	0.70	13.40
PY03673	I-13	NYQEQIENM	9	1000	0.71	53.11
PY03673	I-14	IYSNKRRLI	9	16	0.94	21.72
PY03673	I-15	YIQKNVDTL	9	519	0.95	31.35
PY03673	I-16	VRPFKNNII	9	532	35.68	0.11
PY03673	I-17	EMPIYLNNI	9	269	34.03	0.21
PY03673	I-18	LFPFNDFVL	9	624	29.13	0.48
PY03673	I-19	KKPYFYIGI	9	1346	34.97	0.50
PY03673	I-20	TTPLDNAVI	9	1052	37.90	0.58
PY03673	I-21	VTPEMPIYL	9	266	38.02	0.73
PY03673	I-22	YAPCDKRSI	9	978	11.93	0.81
PY03673	I-23	CTPLNIFTS	9	446	78.89	0.88
PY03673	I-24	VGKFREFYS	9	987	67.19	0.92
PY03832	I-1	YYQTPLSEI	9	1799	0.03	29.16
PY03832	I-2	LYQLTIESI	9	431	0.10	19.70
PY03832	I-3	IYKQNFDSI	9	2897	0.16	14.04
PY03832	I-4	CFLKVRNTI	9	1929	0.17	11.25
PY03832	I-5	KYRIVQNKI	9	2668	0.20	49.20
PY03832	I-6	LYYFDSSNI	9	1191	0.23	22.39
PY03832	I-7	QNHTTFSTI	9	2931	0.31	15.19
PY03832	I-8	FFINSINNI	9	1914	0.33	9.40
PY03832	I-9	SILSAENSI	9	1392	0.38	16.33
PY03832	I-10	AFQTRLDQI	9	1351	0.40	3.48
PY03832	I-11	LRLFMETDI	9	1105	0.44	43.82
PY03832	I-12	TINNSNTYI	9	1556	0.46	26.10
PY03832	I-13	FYFQFFYDI	9	1175	0.47	19.49
PY03832	I-14	SYLKEHKYL	9	630	0.48	84.01
PY03832	I-15	NYPNKDFTF	9	2482	36.72	0.04
PY03832	I-16	IGPSKSQDK	9	1307	75.41	0.06
PY03832	I-17	NGPQMVFQS	9	795	41.36	0.13
PY03832	I-18	NNPNLSNTI	9	1738	2.97	0.18
PY03832	I-19	GDPTCAICA	9	91	96.89	0.28
PY03832	I-20	TGAKIDFLI	9	218	8.46	0.30
PY03832	I-21	HSPLVRFFM	9	517	34.39	0.31
PY03832	I-22	NIPRKTSYI	9	1198	10.89	0.38
PY03832	I-23	VDPKFKQRF	9	249	72.76	0.43
PY03832	I-24	AFPCKHPSI	9	2071	29.81	0.46
PyCSP	I-1	SYVPSAEQI	9	304	0.07	64.51
PyCSP	I-2	QGPGAPQGP	9	139	74.58	0.24
PyCSP	I-3	QGPGAPQEP	9	247	72.32	0.36
PyCSP	I-4	EPPQQPPQQ	9	254	97.28	0.37
PyCSP	I-5	QPPQQPPQQ	9	258	88.69	0.65
PyCSP	I-6	QPPQQPRPQ	9	278	74.44	0.77
PyCSP	I-7	SYVPSAEQIL	10	304	0.26	67.36

Antigen	MHC-peptide	Sequence	Length	Position	H-2 IAd percentile	H-2 IEd percentile
PY01533	II-1	YIVRFLQQRLRIGVN	15	256	0.04	63.90
PY01533	II-2	TSEAKYIVRFLQQRL	15	251	0.40	67.74
PY01533	II-3	LMKKCTVKTGVPVQP	15	436	0.65	60.29
PY01533	II-4	TVKTGVPVQPMLAKP	15	441	1.19	97.50
PY01533	II-5	AHYVWEIKAADLSLS	15	746	1.71	35.30
PY01533	II-6	QIVKDQIMPGVTESI	15	511	1.89	92.55
PY01533	II-7	LQQRLRIGVNSATVL	15	261	1.95	75.83
PY01533	II-8	RSLNWLKVKKDYIEG	15	646	46.88	1.45
PY01533	II-9	RRKLLYSLLRCKEGV	15	586	32.58	1.62
PY01606	II-1	FFGMSMCCQQITMSF	15	481	0.38	85.41
PY01606	II-2	LVTDMVISPHSRYVT	15	336	0.54	74.89
PY01606	II-3	HISSLFVRDPIVVFE	15	606	0.67	78.09
PY01606	II-4	DQLAVIAPLFLAITA	15	506	1.02	84.56
PY01606	II-5	GWRVEFRTPDIQITD	15	731	1.55	75.32
PY01606	II-6	SRYVTLTKNIRKRRG	15	346	22.65	0.54
PY01606	II-7	LEENLFRSSKRDAIL	15	776	28.74	0.82
PY01606	II-8	NEYIQFVKLRCKGEI	15	851	44.33	1.22
PY01606	II-9	VHSYKCNKDRKDEHI	15	31	80.68	1.34
PY01606	II-10	SMTNKKKINEKKKKI	15	411	85.70	1.88
PY03673	II-1	IKSSINKALSILTNN	15	226	1.98	46.85
PY03673	II-2	KKIKLYFASINRKKK	15	751	35.69	0.14
PY03673	II-3	YFASINRKKKKINEI	15	756	63.50	0.26
PY03673	II-4	YKFLKYLKNKKENKI	15	1161	76.55	0.47
PY03673	II-5	VTKYNVLLKKKKFIL	15	936	56.14	0.90
PY03673	II-6	NKGLLKFLNDEKKKY	15	171	91.45	0.92
PY03673	II-7	SNTLYYKFLKYLKNK	15	1156	65.39	0.98
PY03673	II-8	PIYWIKKLEKLKNIN	15	136	38.59	1.25
PY03673	II-9	AQHFFHPLNKKGDIM	15	726	91.74	1.72
PY03832	II-1	TYFKWIINMGAPTAA	15	641	0.26	67.62
PY03832	II-2	IINMGAPTAAASTTA	15	646	0.27	98.46
PY03832	II-3	FDMKALRQSIILLGG	15	2906	0.85	53.36
PY03832	II-4	YWVRNGPQMVFQSNE	15	791	0.98	84.23
PY03832	II-5	EIMRICLDLKYLLY	15	336	1.00	66.42
PY03832	II-6	YWYKYVYLKKIKKKK	15	541	46.62	0.04
PY03832	II-7	RKTSYIKYKKRGKYQ	15	1201	64.70	0.17
PY03832	II-8	DIADEFYFKYKSHHK	15	2281	82.55	0.33
PY03832	II-9	CIDDLYWYKYVYLKK	15	536	36.98	0.37
PY03832	II-10	IIEKRKKGKNKKKLE	15	1876	73.62	0.58
PyCSP	II-1	QILEFVKQISSQLTE	15	311	0.31	92.98
PyCSP	II-2	MKKCTILVVASLLLV	15	1	0.47	94.84
PyCSP	II-3	DSLLPGYGQNKSVQA	15	16	0.90	34.94
PyCSP	II-4	GYGQNKSVQAQRNLN	15	21	1.15	54.46

Summary

Class I 9mers	H-2 Kd	H-2 Dd	Total
PY01533	4	15	19
PY01606	10	13	23
PY03673	15	9	24
PY03832	14	10	24
PyCSP	2	5	7

Class II 15mers	H-2 IAd	H-2 IEd	Total
PY01533	7	2	9
PY01606	5	5	10
PY03673	1	8	9
PY03832	5	5	10
PyCSP	4	0	4
